# Comprehensive metabolites characterization of *Alpinia katsumadai* seeds via a multiplex approach of UHPLC–MS/MS and GC–MS techniques

**DOI:** 10.1038/s41598-025-29831-4

**Published:** 2025-12-09

**Authors:** Nermeen B. Ali, Mai E. Hussein, Mohamed A. Farag

**Affiliations:** Department of Pharmacognosy, Faculty of Pharmacy, Cairo University, El-Kasr El-Aini St, Cairo, 11562 Egypt

**Keywords:** *Alpinia katsumadai* hayata, Zingiberaceae, UHPLC–MS/MS, Molecular networking, Silylated GC–MS, Solid-phase micro-extraction (SPME), Plant sciences, Chemistry

## Abstract

*Alpinia katsumadai* Hayata (Zingiberaceae) is an edible seed valued in traditional medicine for its remarkable therapeutic properties. The seed serves as a fragrant culinary spice, adding a unique flavor to various foods. In this study, three approaches were employed to elucidate seed metabolome including UHPLC-MS/MS-based molecular networking, HS-SPME/GC–MS, and post-silylation GC–MS. A total of 82 secondary metabolites spanning diverse chemical classes were annotated, including the report of two newly annotated catechin-guibourtinidol derivatives and deoxy phloretin-3’-C-hexoside for the first time in *Alpinia* genus, alongside the first hypothesis of calyxins’ class mass fragmentation pattern. HS-SPME/GC–MS analysis led to the detection of 30 aroma determinants dominated by sesquiterpenes (55.1%) and monoterpenes (36.8%). Daucene and α-phellandrene were detected at 274.41 µg/g and 161.25 µg/g, respectively, and to likely account for seed aroma. GC–MS post-silylation dissected 48 metabolites, revealing the abundance of sugars, arylheptanoids, and flavonoids, with sucrose as major sugar (23.81 mg/g), and cardamonin, as major flavonoid. Such comprehensive phytochemical mapping provides critical insights into *A. katsumadai* seed metabolome, offering novel evidence for its health-promoting and culinary qualities.

**Supplementary Information:**

The online version contains supplementary material available at 10.1038/s41598-025-29831-4.

## Introduction

*Alpinia* is one of the foremost genera in the ginger family (Zingiberaceae) that has been used as food spice, flavoring agent, and in ethnomedicine in several countries as China, Japan, and India^1,2^. The genus comprises ca. 250 species that are widely distributed throughout tropical and subtropical regions of the globe^2,3^. Volatile oil, terpenes, phenylpropanoids, diarylheptanoids, and flavonoids are the major classes of chemical constituents commonly found in *Alpinia* species, as reported in several research studies^2,4,5^. Essential oil is a principal component of this genus with a complex chemical profile rich in monoterpenes and sesquiterpenes. It is not only responsible for the characteristic aroma of *Alpinia* but also contributes to a wide range of potential bioactivities, including anti-inflammatory, antimicrobial, cytotoxic, anti-hypertensive, and antioxidant activities, supporting their potential therapeutic applications^1,2,4–6^.

*Alpinia katsumadai* Hayata is an herbaceous species originating from India and is widely cultivated in Southeast Asia, including China^7,8^. Phytochemical analyses of *A. katsumadai* have uncovered a remarkable and impressive spectrum of metabolites, including stilbenes, chalcones, diarylheptanoids, kavalactones, monoterpenes, sesquiterpenes, and flavonoids^9–11^. This distinctive blend of phytochemicals underpins the special therapeutic potential of *A. katsumadai* in both traditional and modern medicine^5,8^. The seeds of *A. katsumadai* (AKS) are documented in the Korean pharmacopeia^5,8^ and are currently regarded as a valuable and important Traditional Chinese Medicine (known as Cao Dou Kou) used in the treatment of numerous ailments, such as emesis and digestive disorders^9,10^. Seeds are characterized by a distinctive warm aroma and a pungent, faintly bitter taste that could be derived from their constituents, especially essential oil content, imparting a distinctive flavor profile^12^.

Culinary and medicinal uses of AKS is attributed for its complex chemical composition, which is marked by the prominence of stilbenes, chalcones, diarylheptanoids, terpenes, and flavonoids, contributing to its distinctive medicinal and culinary properties^5,13^. Recent research highlights that AKS phenolic-rich extract could serve as effective natural preservatives and functional additives in food products through their inhibitory activity against foodborne pathogens such as *Campylobacter jejuni* and *Staphylococcus aureus*, thereby extending product shelf life and enhancing food safety^13^. A wide range of pharmacological properties have been reported for various AKS extracts and their isolated compounds, including anti-emetic, gastroprotective, anti-inflammatory, antioxidant, neuroprotective, antiviral, and anticancer attributes, underscoring their therapeutic potential^7,14–16^. Few studies highlighted the phytoconstituents of AKS that were reviewed in^4,5^. The essential oil composition of AKS is dominated by methyl cinnamate (64.2%) alongside alcohols (7.3%), sesquiterpenes (6.8%), and monoterpenes (5.9%)^10^. In comparison, other studies reported farnesol (14.59 − 21.56%), and 1,8-cineole (18.32–28.20%), as principal constituents in seeds from Liaoning and Yunnan^5,10^. Shell-derived oil showed 1,8-cineole (19.18%),β-pinene (11.76%),terpinen-4-ol (10.42%),α-thujone (10.01%),and p-cymene (9.28%) s the major components^17^. Furthermore, AKS is considered a rich source of diarylheptanoids and flavonoids^11,18^. Diarylheptanoids constitute a broad class of secondary metabolites, dominating in *Alpinia* drugs, especially AKS, known for their diverse biological activities^12,19^. A recent study have reported the isolation of a suite of bioactive compounds including acyclic triterpenoids, an acyclic sesquiterpenoid, an arylheptanoid, and two diarylheptanoids, offering promising leads for the development of novel therapeutics targeting hypercholesterolemia^20^. One of the major bioactive flavonoids in AKS is cardamonin, a chalcone compound that is also reported in cardamom spice and contributes to seeds’ characteristic cardamom-like aroma^21^. Cardamonin is known for its diverse health benefits, including antitumor, antioxidant, and various other medicinal properties^22^. This multifaceted compound not only enhances AKS culinary attributes, but also significantly contributes to its overall medicinal value.

Despite the growing interest in the AKS for its diverse bioactive compounds, a notable gap remains in their thorough chemical characterization. Few studies have conducted detailed metabolomic profiling of this species, and no comprehensive analysis employing multiple complementary analytical techniques has yet been reported.

Driven by the remarkable and distinctive chemical profile previously reported for AKS, alongside the lack of comprehensive chemical exploration of its phytochemical potential, this study presents an in-depth profiling of its phytoconstituents targeting aroma, primary and secondary metabolites. Employing advanced analytical platforms exemplified by UHPLC-MS/MS-based molecular networking, SPME-GC–MS, GC–MS post-silylation, to provide a comprehensive profile of bioactive compounds, and adding to its seed rich chemical makeup for nutraceutical and pharmaceutical development.

UHPLC-high resolution MS/MS led to the detection of a broader range of phytochemicals, while molecular networking maps complex phytochemical relationships, identifying novel compounds and searching bioactive clusters against known libraries. SPME-GC–MS provided the true aroma composition in AKS as cold collection method, whereas, post-silylation GC–MS provided insights into nutritive and low molecular weight secondary metabolites post derivatization. Such a multiplex analytical approach provides unparalleled resolution of AKS’s metabolome, and aid to validate traditional uses of AKS based on such detailed analysis, leading to a paradigm shift from descriptive phytochemistry to functional metabolomics.

## Results and discussion

### Metabolites profiling of AKS via UHPLC-ESI-QTOF-MS/MS-based molecular networking

**Fig. 1:**
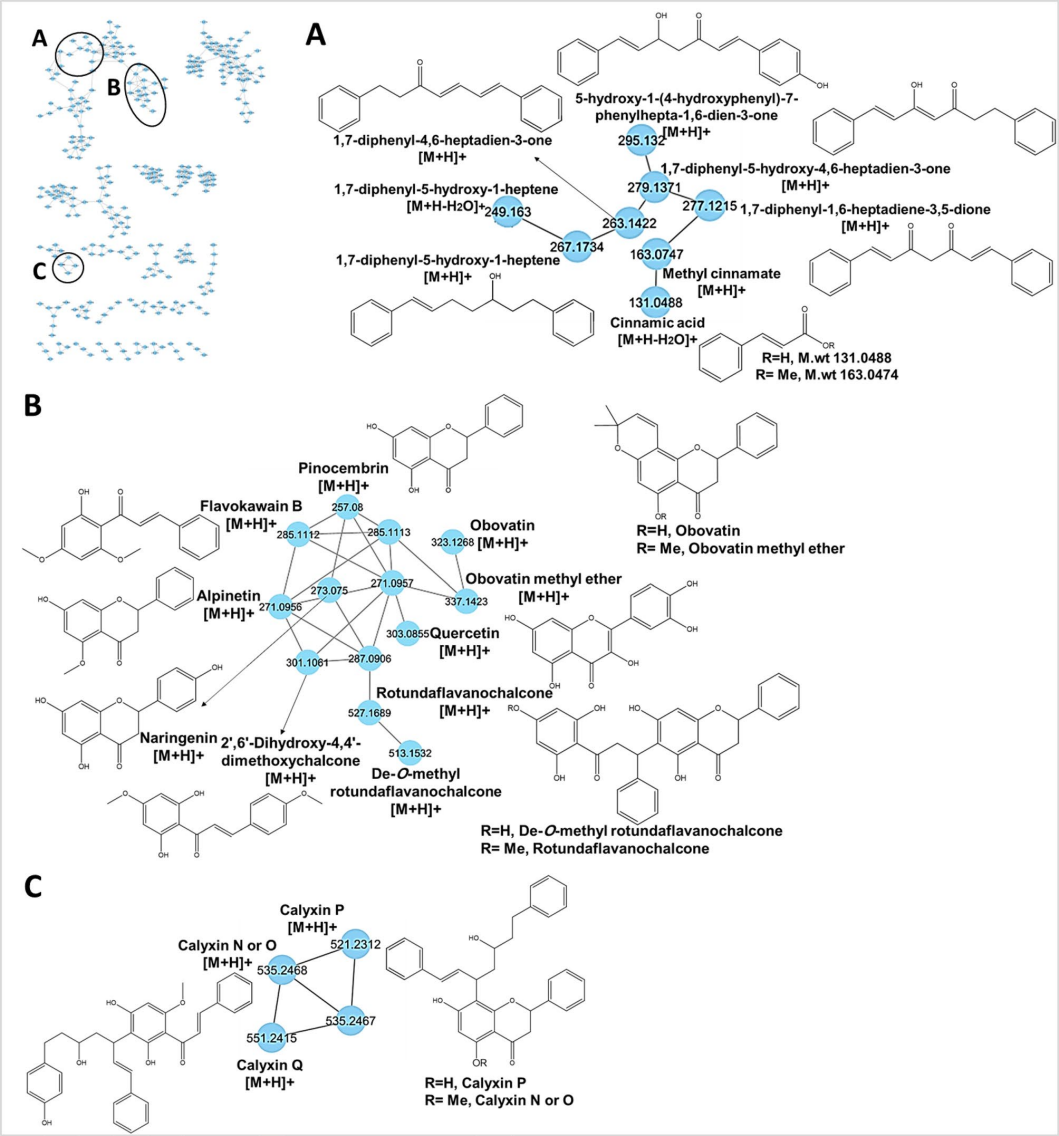
GNPS molecular network of AKS in positive ion mode. The node label represents precursor mass (*m/z*). Cluster **A**, **B**, and **C** represents (diarylheptanoids), (flavanones & flavonochalcones & chalcones) and (calyxins), respectively.

**Fig. 2:**
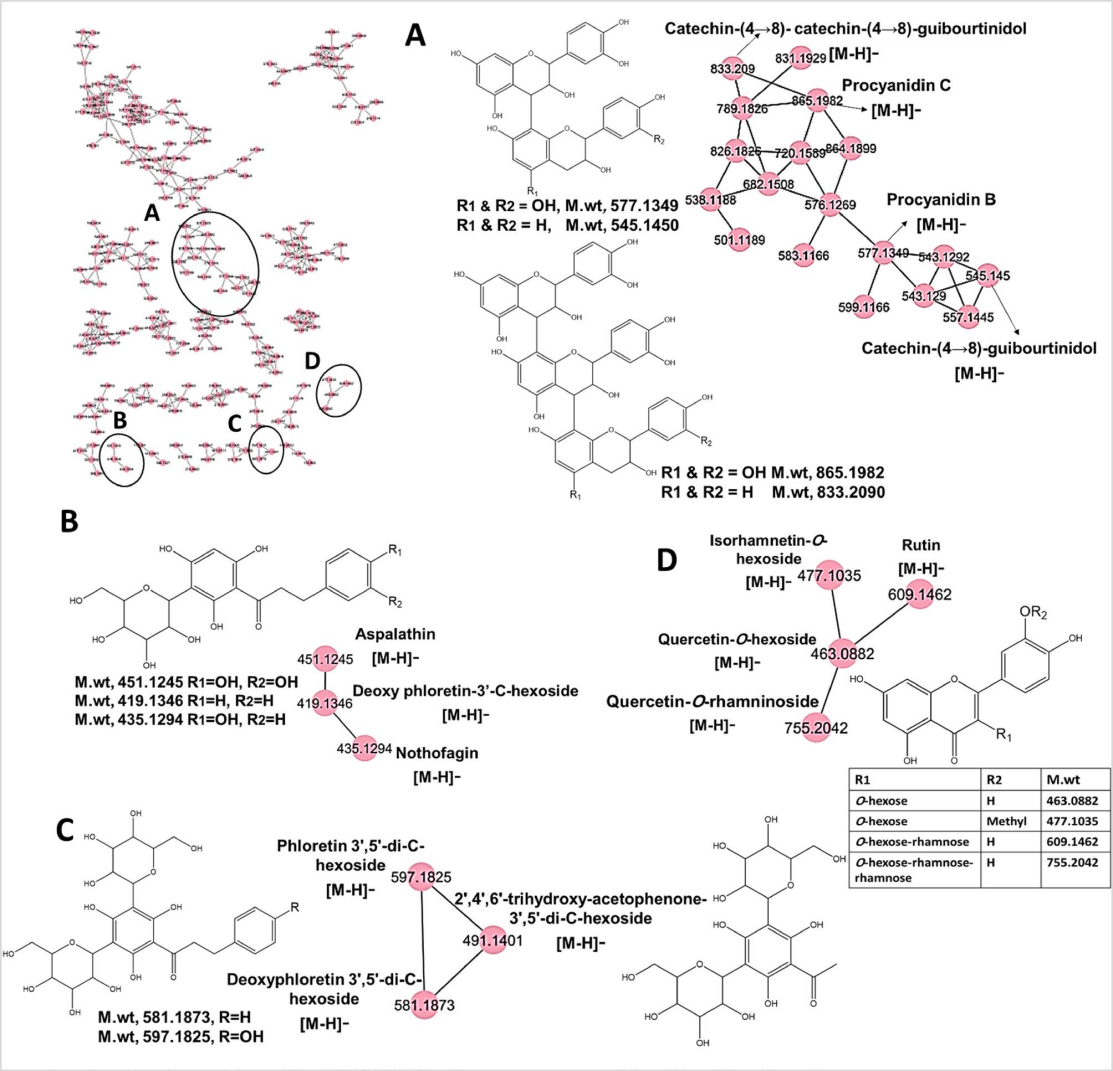
GNPS molecular network of AKS in negative ion mode. The node label represents precursor mass (*m/z*). Cluster **A**, **B**, **C** and **D** represents (proanthocyanidins), (dihydrochalcone mono-C-hexoside), (dihydrochalcone di-C-glycosides) and (flavonol glycosides), respectively.

Metabolites profiling of AKS was carried out in positive and negative modes via UHPLC-ESI-MS/MS-Based Molecular Networking to provide comprehensive metabolome coverage. Representative chromatograms are depicted in (Fig. S1). Metabolites were tentatively identified based on their molecular formulas, retention times and their fragmentation patterns, compared to previous reported data aided with GNPS spectral library search. As listed in Table 1 and 82 metabolites were annotated, belonging to different classes including sugars (L1–L3), amino acids and nitrogenous compounds, (L4–L7), organic acids (L8–L15), phenolic acids (L16–L22), flavonoids (L23–L50), chalcones and dihydrochalcones (L51–L65), calyxins (L66–L68), linear diarylheptanoids (L69–L73), kavalactones (L74–L75), and miscellaneous metabolites (L76–L82). UHPLC-ESI- MS/MS peak numbers are preceded by “L” to be discriminated from GC/MS peaks. This study is the first to assess the phytochemical makeup of AKS holistically using UHPLC-ESI-MS/MS and visualized using molecular networking. Visual analysis of MS/MS data via molecular networking aiding in identification of new metabolites that were tentatively identified for the first time in AKS. Two molecular networks were separately displayed in positive (Fig. 1) and negative (Fig. 2) ionization modes for assignment of compounds as explained in the next subsection for each class in details. All tentatively identified compounds chemical structures are presented in Table S1.

**Table 1:** Identified metabolites detected in AKS using UHPLC-ESI-MS/MS analysis in positive and negative ionization modes.

No.	Rt	Identification	Molecular formula	Mass m/z	Error (ppm)	Adduct		MS/MS fragment (+)	MS/MS fragment (−)	Refs.
*Sugars*										
L1	0.65	Gluconic acid	C_6_H_12_O_7_	195.0501	4.75	[M-H]^−^			87.007, 75.007, 72.992, 71.013, 59.013	^58^
L2	0.66	Glucose/Galactose*	C_6_H_12_O_6_	179.0561	0.07	[M-H]^−^			89.023, 72.992, 71.013, 59.013	^58^
L3	0.69	*O*-Hexosyl-hexose	C_12_H_22_O_11_	341.1087	0.69	[M-H]^−^			179.056, 119.034, 89.023, 71.013, 59.013	^58^
*Amino acids and nitrogenous compounds*										
L4	1.42	Pyroglutamic acid	C_5_H_7_NO_3_	130.0496	2.07		[M + H]^+^	84.045		^59^
L5	2.70	Guanosine	C_10_H_13_N_5_O_5_	282.0843	0.33	[M-H]^−^			150.041, 133.015, 126.030, 108.019, 107.035	^58^
L6	3.18	Phenylalanine*	C_9_H_11_NO_2_	166.0859	2.14		[M + H]^+^	120.081, 103.054, 93.070		^59^
L7	4.23	Tryptophan*	C_11_H_12_N_2_O_2_	203.0818	3.95	[M-H]^−^			142.065, 116.049	^60^
*Organic acids*										
L8	0.66	Xylonic acid	C_5_H_10_O_6_	165.0397	4.61	[M-H]^−^			87.008, 75.007, 72.992, 71.013, 59.013	^58^
L9	0.67	Threonic acid	C_4_H_8_O_5_	135.0297	1.46	[M-H]^−^			75.008, 72.992, 71.013, 59.013	^58^
L10	0.70	Fumaric acid	C_4_H_4_O_4_	115.0032	4.19	[M-H]^−^			71.012	^61^
L11	0.70	Malic acid*	C_4_H_6_O_5_	133.0141	1.10	[M-H]^−^			115.002, 72.992, 71.013	^58^
L12	0.70	Aconitic acid	C_6_H_6_O_6_	173.0092	−0.22	[M-H]^−^			111.008, 85.028	^61^
L13	0.72	Citric acid*	C_6_H_8_O_7_	191.0189	4.33	[M-H]^−^			111.008, 87.006, 85.028, 67.018, 59.013, 57.033	^58^
L14	6.64	Suberic acid	C_8_H_14_O_4_	173.0815	2.50	[M-H]^−^			129.091, 111.080	^62^
L15	8.01	Azelaic acid	C_9_H_16_O_4_	187.0967	4.72	[M-H]^−^			125.096, 97.065	^61^
*Phenolic acids*										
L16	3.74	Protocatechuic acid*	C_7_H_6_O_4_	153.0192	0.86	[M-H]^−^			109.028, 108.021, 91.018	^61^
L17	4.27	Vanillic acid hexoside*	C_14_H_18_O_9_	329.0876	0.63	[M-H]^−^			209.045, 167.034, 123.044	^63^
L18	4.58	Hydroxybenzoic acid*	C_7_H_6_O_3_	137.0242	1.59	[M-H]^−^			119.013, 108.020, 93.033	^61^
L19	6.43	Coumaric acid*	C_9_H_8_O_3_	163.0398	1.64	[M-H]^−^			119.049, 93.033	^63^
L20	6.53	Vanillic acid*	C_8_H_8_O_4_	167.0350	−0.11	[M-H]^−^			152.010, 123.008,108.020, 91.018	^61^
L21	7.13	Ferulic acid*	C_10_H_10_O_4_	193.0498	4.31	[M-H]^−^			178.027, 134.036, 133.028, 89.039	^63^
L22	13.57	Cinnamic acid*	C_9_H_8_O_2_	131.0488	1.20		[M + H–H_2_O]^+^	103.054, 95.094, 77.390		^64^
*Flavonoids*										
*Flavonols*										
L23	6.14	Quercetin-*O*-rhamninoside (Flavovilloside)	C_33_H_40_O_20_	755.2042	−0.24	[M-H]^−^	[M + H]^+^	611.157, 465.101, 303.049	301.035, 300.028, 255.982, 178.998	^65^
				757.2172	1.81					
L24	6.72	Typhaneoside*	C_34_H_42_O_20_	769.2201	−0.56	[M-H]^−^			706.757, 530.627, 315.054, 314.045	^66^
L25	6.75	Rutin*	C_27_H_30_O_16_	609.1462	−0.15	[M-H]^−^	[M + H]^+^	303.049	301.035, 300.025, 151.003	^66^
				611.1595	1.90					
L26	6.95	Quercetin-*O*-hexoside*	C_21_H_20_O_12_	463.0882	0.00	[M-H]^−^			301.035, 300.028, 178.998, 151.003	^58^
L27	7.46	Isorhamnetin-*O*-rutinoside*	C_28_H_32_O_16_	623.1618	−0.07	[M-H]^−^	[M + H]^+^	317.065	315.050, 314.044, 151.179	^66^
				625.1750	2.10					
L28	7.68	Isorhamnetin-*O*-hexoside*	C_22_H_22_O_12_	477.1035	0.73	[M-H]^−^			315.053, 314.043, 151.003	^66^
L29	9.44	Quercetin*	C_15_H_10_O_7_	301.0351	0.92	[M-H]^−^	[M + H]^+^	257.043, 229.049, 153.018	273.039, 178.998, 151.003, 121.028	^66^
				303.0491	2.74					
L30	10.74	Kaempferol*	C_15_H_10_O_6_	285.0402	0.92	[M-H]^−^			151.003, 185.061, 107.012, 65.362	^66^
L31	12.79	Galangin*	C_15_H_10_O_5_	269.0451	1.66	[M-H]-			223.039, 213.055, 195.048, 171.043, 169.065, 143.049	^67^
*Flavanone*										
L32	8.19	Sakuranetin	C_16_H_14_O_5_	285.0767	0.52	[M-H]^−^	[M + H]^+^	167.033, 131.049, 103.054	119.049, 165.018	^67^
				287.0907	2.44					
L33	9.38	Pinocembrin-*O*- hexoside	C_21_H_22_O_9_	417.1189	0.49	[M-H]^−^			255.066, 213.056,177.018, 151.003	^68^
L34	10.37	Naringenin*	C_15_H_12_O_5_	271.0610	0.73	[M-H]^−^	[M + H]^+^	153.018, 147.044 119.049	187.040, 151.003, 119.049	^66^
				273.0750	2.75					
L35	11.01	Violanone	C_17_H_16_O_6_	315.0871	0.99	[M-H]^−^			300.028, 268.038	^69^
L36	12.73	Pinocembrin*	C_15_H_12_O_4_	255.0659	1.50	[M-H]^−^	[M + H]^+^	215.0708, 153.018, 131.049, 103.054, 91.055	213.055, 211.076, 187.076, 171.044, 151.003	^23^
				257.0800	3.25					
L37	13.33	Sativanone	C_17_H_16_O_5_	299.0911	4.67	[M-H]^−^			284.069, 256.078	^69^
L38	13.42	Alpinetin*	C_16_H_14_O_4_	271.0956	3.27		[M + H]^+^	167.033, 131.049, 103.054, 91.054		^23^
L39	14.12	Obovatin methyl ether	C_21_H_20_O_4_	337.1423	3.37		[M + H]^+^	233.080, 215.070, 205.058, 131.049, 103.054		^24^
L40	14.40	Obovatin	C_20_H_18_O_4_	323.1268	3.05		[M + H]^+^	219.065, 103.054		^25^
*Flavanonols*										
L41	7.13	Taxifolin (Dihydroquercetin)	C_15_H_12_O_7_	303.0509	0.42	[M-H]^−^			153.019, 125.023	^67^
L42	8.32	Dihydroisorhamnetin	C_16_H_14_O_7_	317.0664	0.87	[M-H]^−^			192.042, 178.997, 152.011, 125.023	^67^
L43	10.74	Dihydrokaempferide	C_16_H_14_O_6_	301.0714	1.20	[M-H]^−^			283.061, 151.003	^69^
L44	12.87	Pinobanksin-*O*-acetate*	C_17_H_14_O_6_	313.0715	0.84	[M-H]^−^			271.062, 253.050, 225.054, 209.060, 197.061	^67^
*Proanthocyanidin*										
L45	4.56	Catechin-(4→8)-catechin (Procyanidin B)*	C_30_H_26_O_12_	577.1349	0.43	[M-H]^−^			451.105, 425.088, 407.07, 289.072, 287.056, 245.082, 125.023	^70^
L46	4.96	Catechin*	C_15_H_14_O_6_	289.0716	0.56	[M-H]^−^			245.082, 203.071, 179.035, 165.018, 151.039, 137.023, 125.023, 109.028	^70^
L47	5.00	Catechin-(4→8)- catechin-(4→8)-catechin (Procyanidin C)	C_45_H_38_O_18_	865.1982	0.39	[M-H]^−^			713.152, 577.135, 575.120, 451.102, 425.089, 407.077, 289.072, 287.056	^70^
L48	5.05	Cinnamtannin A2	C_60_H_50_O_24_	1155.2737	2.41		[M + H]^+^	985.198, 865.182, 579.149		^70^
L49	10.98	Catechin-(4→8)- catechin-(4→8)-guibourtinidol**	C_45_H_38_O_16_	833.2090	0.19	[M-H]^−^			681.158, 545.142, 419.114, 393.098, 287.056, 257.082 161.024, 125.023	
L50	11.56	Catechin-(4→8)-guibourtinidol**	C_30_H_26_O_10_	545.1450	0.59	[M-H]^−^			419.113, 393.097, 287.055, 257.082, 161.024, 125.023	
*Chalcones and dihydrochalcones*										
L51	3.43	Aspalathin	C_21_H_24_O_11_	451.1245	0.19	[M-H]-			361.093, 331.082, 289.817, 167.034, 125.023	^71^
L52	7.03	Phloretin-3’,5’-di-C-hexoside	C_27_H_34_O_15_	597.1825	−0.01	[M-H]^−^			477.138, 417.120, 387.108, 357.099	^72^
L53	7.45	Nothofagin	C_21_H_24_O_10_	435.1294	0.62	[M-H]^−^			345.098, 315.088, 273.077, 167.034, 125.023	^73^
L54	8.82	Deoxy phloretin-3’,5’-di-C-hexoside (2’,4’,6’-trihydroxy-3’,5’-di-C-hexoside-dihydrochalcone)	C_27_H_34_O_14_	581.1873	0.48	[M-H]^−^			461.146, 401.124, 371.113, 341.103	^74^
L55	9.80	Deoxy phloretin-3’-C-hexoside** (2’,4’,6’-trihydroxy-3’-C-hexoside dihydro-chalcone)	C_21_H_24_O_9_	419.1346	0.37	[M-H]^−^			329.103, 299.092, 257.082, 167.034, 125.023	
L56	10.44	Phloretin	C_15_H_14_O_5_	273.0767	0.54	[M-H]^−^			167.034, 123.044, 119.049, 81.033	^71^
L57	11.93	Sakuranetin Dihydrochalcone	C_16_H_16_O_5_	287.0922	1.04	[M-H]^−^			193.050,181.050, 152.011, 139.039, 124.016, 93.033	^67^
L58	12.73	Pinocembrin dihydrochalcone	C_15_H_14_O_4_	257.0815	1.68	[M-H]^−^			213.091, 171.081, 169.102, 156.057, 122.036	^67^
L59	12.90	Flavokawain B* (Flavokavain B)	C_17_H_16_O_4_	285.1112	3.28		[M + H]^+^	181.049, 131.049		^75^
L60	13.24	Dihydroxy-dihydrochalcone	C_15_H_14_O_3_	241.0865	2.15	[M-H]^−^			197.097, 121.028	^76^
L61	13.32	2’,6’-dihydroxy-4,4’-dimethoxychalcone	C_17_H_16_O_5_	301.1077	1.49		[M + H]^+^	167.033, 161.059		^77^
L62	13.41	De-*O*-methyl rotundaflavanochalcone	C_30_H_24_O_8_	513.1532	2.33		[M + H]^+^	387.122, 345.112, 257.080, 153.018		^40^
L63	13.42	Cardamonin*	C_16_H_14_O_4_	269.0816	1.24	[M-H]^−^			254.058, 226.063, 165.018, 121.998	^23^
L64	13.52	Dihydroxy-methoxy dihydrochalcone	C_16_H_16_O_4_	271.0973	1.04	[M-H]^−^			210.145, 152.011, 165.018, 124.016	^67^
L65	13.95	Rotundaflavano-chalcone	C_31_H_26_O_8_	527.1689	2.17		[M + H]^+^	387.122, 345.111, 271.096, 167.033		^40^
*Calyxins (chalcone/flavanone-diarylheptanoids)*										
L66	14.05	Calyxin N/O*	C_35_H_34_O_5_	535.2467	2.24		[M + H]^+^	517.237, 413.174, 283.095, 255.064, 179.033, 131.049, 117.070, 91.055		^41^
L67	14.24	Calyxin Q*	C_35_H_34_O_6_	551.2415	2.39		[M + H]^+^	533.232, 413.175, 283.098, 255.054, 179.034, 131.049, 117.070, 91.055		^41^
L68	14.65	Calyxin P*	C_34_H_32_O_5_	521.2312	1.82		[M + H]^+^	503.220, 399.159, 345.111, 269.080, 165.018, 131.085, 117.070, 91.055		^41^
*Linear diarylheptanoids*										
L69	13.56	1,7-diphenyl-5-hydroxy-1-heptene*	C_19_H_22_O	267.1734 249.1630	3.52		[M + H]^+^ [M + H–H_2_O]^+^	131.085, 117.070, 105.070, 91.055		^78^
L70	13.95	5-hydroxy-1-(4-hydroxyphenyl)−7-phenylhepta-1,6-dien-3-one	C_19_H_18_O_3_	295.1320	2.95		[M + H]^+^	147.04, 131.049, 107.049, 91.055		^45^
L71	14.53	1,7-diphenyl-4,6-heptadien-3-one* (Alnustone)	C_19_H_18_O	263.1422	3.20		[M + H]^+^	157.065, 105.070, 91.055		^23^
L72	14.94	1,7-diphenyl-5-hydroxy-4,6-heptadien-3-one*	C_19_H_18_O_2_	279.1371	3.07		[M + H]^+^	131.049, 105.070, 91.055		^78^
L73	15.01	1,7-diphenyl-1,6-heptadiene-3,5-dione	C_19_H_16_O_2_	277.1215	2.91		[M + H]^+^	173.060, 131.049, 103.054, 91.054		^79,80^
*Kavalactones*										
L74	11.28	4-hydroxy-6-styryl-2 H-pyran-2-one	C_13_H_10_O_3_	213.0549	3.12	[M-H]^−^	[M + H]^+^	187.075, 169.064, 141.070, 131.049, 115.054, 103.054	171.044, 169.065, 141.070, 139.054	^81^
				215.0696						
L75	12.84	Desmethoxy yangonin (5,6-dehydrokawain)*	C_14_H_12_O_3_	229.0853	2.71		[M + H]^+^	201.091, 183.080, 141.069, 131.049, 103.054		^82^
*Miscellaneous*										
L76	0.61	Cinnamaldehyde*	C_9_H_8_O	131.0504	−1.23	[M-H]^−^			114.019, 113.035, 72.008, 70.028, 71.024, 71.013, 70.029, 58.029	^58^
L77	0.69	Hydroxy methyl-furaldeyde	C_6_H_6_O_3_	127.0388	1.34		[M + H]^+^	109.029, 81.034, 53.034		^59^
L78	4.72	2ʹ,4ʹ,6ʹ-trihydroxy-acetophenone-3ʹ,5ʹ-di-C-hexoside	C_20_H_28_O_14_	491.1401	1.08	[M-H]^−^			371.097, 311.077, 281.067, 251.056	^72^
L79	5.63	Hydroxybenzaldehyde*	C_7_H_6_O_2_	121.0293	1.68	[M-H]^−^			108.020, 92.025	^61^
L80	9.69	Syringaldehyde*	C_9_H_10_O_4_	181.0498	4.60	[M-H]^−^			166.026, 151.023, 138.031	^83^
L81	12.71	Methyl cinnamate*	C_10_H_10_O_2_	163.0747	4.02		[M + H]^+^	131.049, 121.065, 103.054		^84^
L82	11.87	Syringenin (sinapyl alcohol)	C_11_H_14_O_4_	209.0811	3.98	[M-H]^−^			165.091, 125.060, 123.080, 122.036	^67^

*Previously identified in *Alpinia* genus^4,5,33,85–90^; **new putative metabolites in *Alpinia* genus.

#### Identification of flavonoids

The tentatively identified flavonoids belonged to 4 main subclasses namely, flavonols, flavanone, flavanonols, proanthocyanidins and their corresponding glycosides. Flavonoids generally have the framework C_6_-C_3_-C_6_ that mostly undergo Retro Diels-Alder (RDA) cleavage^23^. Flavonol glycosides represented by quercetin-*O*-rhamninoside (L23), rutin (L25), quercetin-*O*-hexoside (L26), isorhamnetin-*O*-hexoside (L28) were clustered in cluster (D) in negative mode (Fig. 2), exhibiting distinctive losses of 454, 308 and 162 Da corresponding to the cleavage of rhamninoside rutinoside and hexoside moieties, respectively.

Noticeably, cluster (B) in positive mode (Fig. 1) characterized by presence of 5 flavanones; naringenin (L34), pinocembrin (L36), alpinetin (L38), obovatin methyl ether (L39), obovatin (L40). Obovatin and its methyl ether derivative were previously isolated in genera *Tephrosia* and *Dalea* (family Fabaceae)^24,25^. Mass fragmentation of flavanones typically yields^1,3^A^+^ and^1,3^B^+^ fragment ions through RDA fragmentation as base peak. Besides, flavanone itself undergoes cleavage to yield^1,4^B^+^ fragment ions (Fig. S2)^26,27^. Naringenin and pinocembrin showed the same^1,3^A^+^ fragment ion at *m/z* 153.018, but differing in^1,3^B^+^ and ^1,4^B^+^ fragment ions due to the presence of hydroxyl group on ring B in naringenin, yielding fragment ions that differ by 16 Da (Fig. S2A and B). Likewise, alpinetin showed similar fragment ions^1,3^B^+^ and ^1,4^B^+^ at *m/z* 103.054 and 131.049, respectively, with pinocembrin, but differ in ^1,3^A^+^ fragment ion at *m/z*167.033 due to the presence of 5-methoxy group on ring A in alpinetin (Fig. S2C). Besides ^1,3^,A^+^ fragment ions at *m/z* 233.080 and 219.065 for obovatin methyl ether and obovatin indicated the presence of the methyl group (Fig. S2D and E).

Proanthocyanidins (PACs), condensed tannins, are oligomers and polymers consisting of flavan-3-ol monomeric units^28^. Herein, identified proanthocyanidins have the C4→C8 linkages. Catechin represents the basic common building block of proanthocyanidin. Catechin (L46) displayed fragment ion at *m/z* 245.082 from the loss of 44 Da (CH_2_ = CH-OH). Fragment ions at *m/z* 179.035 and 109.028 were detected due to the loss of dihydroxybenzene moiety, loss of ring B. Heterocyclic ring fission (HRF) led to the formation of fragment ions at *m/z* 165.018 and 125.023. retro-Diels–Alder (RDA) cleavage of ring C was characterized by presence of fragment ions at *m/z* 151.039 and 137.023 (Table 1)^29^. Two PACs dimer type B were detected represented by catechin-(4→8)-catechin (procyanidin B) (L45) and catechin-(4→8)-guibourtinidol (L50), Two PACs trimer type B namely, catechin-(4→8)-catechin-(4→8)-catechin (procyanidin C) (L47) and catechin-(4→8)-catechin − (4→8)-guibourtinidol (L49) were identified and clustered in cluster (A) in negative mode (Table 1; Fig. 2). The main fragmentation mechanisms of PACs can be explained by RDA, quinine methide (QM), and HRF fragmentation pathways^28,30,31^. Their MS/MS chromatograms were depicted in (Fig. S3). In general, MS/MS of PACs dimer and trimer type B showed the characteristic fragment ions differing by 32 Da as they distinguished in terminal monomer, guibourtinidol differs from catechin by 32 Da, this was further confirmed by QM cleavage fragment ions at *m/z* 287.056 and 257.082 indicating the presence of catechin and guibourtinidol moieties, respectively (Fig. S4). Interestingly, two new PACs (L49 and L50) were tentatively identified for the first time in *Alpinia* genus based on their first postulated fragmentation patterns (Fig. S3 and S4). The pronounced biological activities of AKS are strongly attributable to its richness in phenolic metabolites which are well recognized for their anti-inflammatory, antimicrobial, antioxidant and anticancer, etc. activities^32^.

#### Identification of chalcones and dihydrochalcones

UHPLC-ESI-MS/MS based molecular networking in positive and negative modes revealed the abundance of chalcone and dihydrochalcones, being detected in clusters (B) in positive mode (Fig. 1) and clusters (B&C) in negative mode (Fig. 2). Among the tentatively identified chalcones and dihydrochalcones in the present study, (L59) flavokawain B and (L63) cardamonin have been previously reported in *Alpinia* genus^4,5,33^, Chalcones and dihydrochalcones have inhibitory activities on enzymes, anti-inflammatory, antioxidant, antibacterial, anticancer, antifungal, antimalarial, anti-filarial and antiprotozoal activity^34,35^. Main fragmentation of chalcones is derived from α-cleavage mechanism leading to the loss of phenyl or styryl radical. In cluster (B) in positive mode (Fig. 1), flavokawain b (L59) and 2’,6’-dihydroxy-4,4’-dimethoxychalcone (L61) showed characteristic fragment ions at *m/z* 181.049 and 167.033, respectively due to the loss of substituted styryl radical and fragment ions at *m/z* 131.049 and 161.059, due to loss of substituted phenyl radical^36,37^ (Fig. S5).

Clusters (B&C) in negative mode (Fig. 2) mainly represented dihydrochalcones-C-glycosides. In C-glycosides, cleavage of sugar moiety, hexose, showed the loss of neutral fragments 120 Da, representing cleavage of 1’→2’ linkage versus 90 Da representing the cleavage of 1’→3’ linkage^38,39^. Cluster (B, Fig. 2) represented dihydrochalcone mono-C-hexoside; aspalathin (L51), nothofagin (L53), and deoxy phloretin-3’-C-hexoside (L55) that yielded fragment ions at *m/z* 361.093, 345.098, 329.103 ([M-H-90]^−^) and *m/z* 331.082, 315.088, 299.092 ([M-H-120]^−^), respectively. Interestingly, fragment ions at *m/z* 289.072, 273.077, 257.082 ([M-H-162]^−^), loss of six-member ring sugar moiety, were also observed^38^ (Fig. S6). Additionally, nothofagin (L53), and deoxy phloretin-3’-C-hexoside (L55) yielded fragment ions at *m/z* 167.034 and 125.023, resulting from β- and α- cleavages of the carbonyl group, respectively (Fig. S6B and C). It is noteworthy that deoxy phloretin-3’-C-hexoside (L55) is a putative compound and the first time to be identified in *Alpinia* genus based on its mass fragmentation.

On the other hand, cluster (C, Fig. 2) exhibited two dihydrochalcone di-C-glycosides represented by phloretin 3’,5’-di-C-hexoside (L52), deoxy phloretin 3’,5’-di-C-hexoside (L54). They were clustered with 2ʹ,4ʹ,6ʹ-trihydroxy-acetophenone-3ʹ,5ʹ-di-C-hexoside (L78) to produce characteristic fragments ions at *m/z* 477.138, 461.146, 371.097 ([M-H-120]^−^), *m/z* 387.108, 371.113, 281.067 ([M-H-210]^−^) and *m/z* 357.099, 341.103, 251.056 ([M-H-240]^−^), respectively, and inferring that peaks L52, 54 and L78 belonged to di-C-glycosides (Fig. S7).

Other dihydrochalcones appearing in positive ion mode, derived network included de-*O*-methyl rotundaflavanochalcone (L62) and rotundaflavanochalcone (L65), flavonochalcones, to encompass a flavanone connected to a dihydrochalcone moiety via a C–C bond, previously isolated from *Boesenbergia rotunda* genus (family Zingiberaceae)^40^. They appeared in cluster (B) in positive mode (Fig. 1). The MS/MS spectrum of both compounds showed the same fragment ion at *m/z* 387.122 due to α cleavage of carbonyl group in the dihydrochalcone moiety leading to loss of phenyl radical. Whilst, they differ in phenyl group substituents leading to the formation of fragment ions at *m/z* 153.018 and 167.033, respectively (Fig. S8). Moreover, C-C glycosidic bond cleavage led to fragment ions at *m/z* 257.080 and 271.096, respectively (Fig. S8) corresponding to dihydrochalcone and flavanone moieties. This is the first time that a fragmentation pattern mechanism of those compounds has been proposed in the literature.

#### Identification of calyxins (chalcone/flavanone-diarylheptanoids)

Various calyxins were previously reported from *Alpinia* genus^4,5^, to exert various biological activities relevant to antidiabetic, antibiotic, antiproliferative, and vasodilative activities^5,41^. Herein, the identified calyxins were clustered in cluster (C) in positive mode (Fig. 1). They consist of pinocembrin (calyxin N/O isomers (L66), and calyxin P (L68) or de-*O*-methyl flavokawain B (calyxin Q, (L67)) attached with diarylheptanoid, mostly identified based on their RDA fragmentation pathway in ring C followed by the elimination of side chain. The MS/MS spectrums of calyxins are similar to each other due to similarity in structures, calyxin P is a demethylated derivative of calyxin N/O, yielding fragment ions that differ from each other by 14 Da, indicating the presence of a methyl group in calyxin N and O (Fig. S9A andC). Whilst, MS/MS spectrum of calyxin Q was identical to that of calyxin N/O, likely due to isomerization of chalcone into their corresponding flavanones^42,43^ (Fig. S9B). It should be noted that this is the first suggested fragmentation pattern of calyxins (Fig. S10). Additionally, fragment ions at *m/z* 147.044, 131.049, 117.070 and 91.055, resulting from mass fragmentation of diarylheptanoid moiety, aided in identification.

#### Identification of diarylheptanoids

Diarylheptanoids are mostly reported from *Alpinia* genus^4,5^, and unique constituents of the Zingiberaceae family to impart several health effects, including hepatoprotective, anticancer, antioxidant, and melanogenesis activities, besides their nutraceutical applications as organoleptic additives in foods^44^. They were identified in cluster (A) in positive mode (Fig. 1). Five diarylheptanoids were detected, represented by 1,7-diphenyl-5-hydroxy-1-heptene (L69), 5-hydroxy-1-(4-hydroxyphenyl)−7-phenylhepta-1,6-dien-3-one (L70), 1,7-diphenyl-4,6-heptadien-3-one (L71), 1,7-diphenyl-5-hydroxy-4,6-heptadien-3-one (L72), 1,7-diphenyl-1,6-heptadiene-3,5-dione (73). (L70) and (L73) were previously detected in turmeric, *Curcuma longa*, (family Zingiberaceae)^45^ and in *Eugenia jambos* (family Myrtaceae)^46^, respectively. Diarylheptanoids encompass two phenyl groups joined by a 7-carbon chain (heptane) and have various substituents and undergo the intermolecular cleavage of the tautomeric isomers, followed by the cleavage of the heptanoid chain between the carbonyl and methylene groups^47,48^. The mass fragmentation spectrum of identified diarylheptanoids is presented in Fig. S11.

### SPME/GC–MS analysis of AKS

**Fig. 3:**
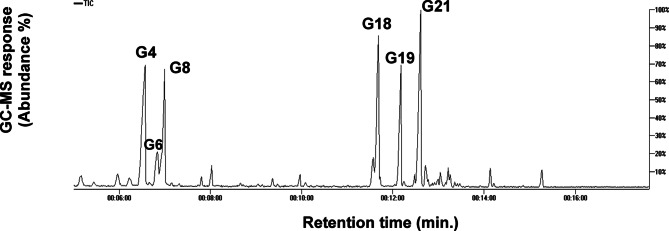
Representative GC–MS chromatogram of AKS for its volatiles analyzed by SPME coupled to GC–MS. The corresponding volatile names for each major peak follow that listed in Table 2. **G4**: α-Phellandrene, **G6**: p-Cymene, **G8**: 1,8 Cineole, **G18**: Daucene, **G19**: β-Caryophyllene, and **G21**: α-Humulene.

Essential oil is regarded as a key component of *Alpinia* taxa, primarily consisting of various terpenoids^4^, to contribute to its culinary uses and further health benefits. This study explored the aroma profile of AKS using SPME/GC–MS analysis. GC–MS peak numbers are preceded by “G”. A total of 30 different volatile constituents were identified and categorized into various classes, with monoterpene and sesquiterpene hydrocarbons most abundant detected at 36.8% and 55.1% of the total volatiles, respectively. A detailed summary of the identified compounds and their absolute quantities (µg/g) is presented in Table 2, while Fig. 3 illustrates GC–MS chromatogram.

**Table 2:** Absolute quantification (µg/g) of volatiles of AKS identified using GC–MS analysis. The values are expressed as average ± st. dev. (*n* = 3).

Peak #	Average Rt (min)	Calculated KI	Metabolite	µg/g ± st. dev.
*Monoterpene hydrocarbons*				
1	5.1492	796	α-Thujene	18.1 ± 3.56
2	5.95	846	Sabinene	19.1 ± 4.14
3	6.2317	863	β-Myrcene*	29.53 ± 11.68
4	6.5958	885	α-Phellandrene	161.25 ± 27.49
5	6.6808	890	Isoterpinolene	0.27 ± 0.34
6	6.8733	902	p-Cymene*	101.25 ± 19.21
7	6.965	908	D-Limonene*	24.03 ± 12.22
9	7.1517	919	β-cis-Ocimene	1.31 ± 1.86
11	8.0417	974	α-Ocimene	33.37 ± 15.05
*Oxides*				
8	7.0208	911	1,8 Cineole*	86.27 ± 23.84
*Ketones*				
10	7.8025	959	L-Fenchone	19.2 ± 7.85
12	8.6575	1013	Camphor*	1.03 ± 0.49
16	9.9642	1103	Benzylacetone	23.02 ± 7.84
*Alcohols*				
14	9.365	1062	Ocimenol	8.66 ± 4.8
*Sesquiterpene hydrocarbons*				
18	11.7042	1238	Daucene	274.41 ± 80.35
19	12.1875	1278	β-Caryophyllene*	62.05 ± 17.06
21	12.6375	1316	α-Humulene	197.56 ± 23.09
22	12.7233	1323	Bicyclosesquiphellandrene	25.43 ± 8.47
23	12.7833	1328	α-Muurolene	1.01 ± 1.43
26	13.2142	1366	Bicyclosesquiphellandrene isomer	9.27 ± 1.12
28	14.1317	1450	Germacrene D	10.54 ± 2.56
*Unknowns*				
13	9.0375	1039	Unknown	4.71 ± 1.86
15	9.4717	1069	Unknown	1.24 ± 0.13
17	10.08	1112	Unknown	0.49 ± 0.27
20	12.4983	1303	Unknown	2.16 ± 1.36
24	12.9892	1347	Unknown	0.26 ± 0.24
25	13.0483	1352	Unknown	8.58 ± 2.51
27	13.255	1370	Unknown	12.18 ± 5.49
29	14.2075	1457	Unknown	0.29 ± 0.21
30	15.2558	1551	Unknown	3.51 ± 0.52

Asterisk denotes peaks confirmed by using the standard.

#### Sesquiterpene hydrocarbons

Sesquiterpene hydrocarbons constituted the major class in AKS, with 7 peaks. Daucene (G18) represented the major form at highest level of 274.41 µg/g, followed by α-humulene (G21) and β-caryophyllene (G19) at 197.56 µg/g and 62.05 µg/g, respectively. α-Humulene and β-caryophyllene are key odor-active compounds in *A. katsumadai* seed oil (AKSO), and their high abundance may contribute significantly to its woody and spicy aroma^49^. Other sesquiterpene hydrocarbons detected at much lower levels included bicyclosesquiphellandrene (G22), α-muurolene (G23) and germacrene D (G28).

#### Monoterpene hydrocarbons

Compared to sesquiterpenes, monoterpene hydrocarbons accounted for the second most prevalent class of volatile compounds in AKS. A total of 9 monoterpene hydrocarbons were detected in AKSO. In contrast to previous reports^4^, our findings show major forms included α-phellandrene (G4) at 161.25 µg/g of potential aroma and used in pharmaceutical, food, and cosmetic industries due to its pleasant scent^50^. The second most abundant volatile was p-cymene (G6) at 101.25 µg/g. Other identified monoterpene hydrocarbons included α-Thujene (G1), sabinene (G2), β-myrcene (G3), isoterpinolene (G5), D-limonene (G7), β-cis-ocimene (G9), and α-ocimene (G11) that were detected at lower levels.

#### Ketones

Ketones accounted for 4.04% of the total volatiles detected in AKS. Among them, L-fenchone (G10), noted for its herbaceous and fragrance-like aroma^51,52^, was detected at a concentration of 19.2 µg/g. Other identified ketones include camphor (G12) and benzylacetone (G16), which were detected at 1.03 and 23.02 µg/g, respectively.

#### Alcohols and oxides

Ocimenol (G14) was the only identified alcohol found at a trace amount of 8.66 µg/g in AKS. whereas 1,8 cineole (G8), a monoterpene oxide, was detected at a relatively high level of 86.27 µg/g compared to other volatile components, which is consistent with findings from previous studies^5^.

### GC–MS profiling of AKS metabolites post-silylation

**Fig. 4:**
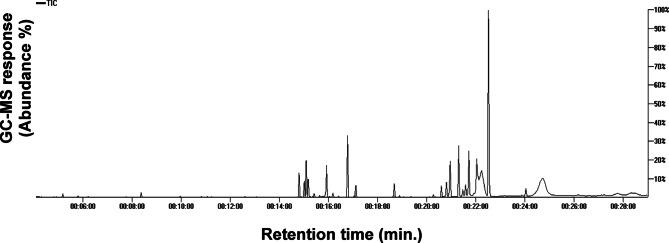
Representative GC–MS chromatogram of AKS for silylated primary metabolites. The corresponding compound numbers for peaks follow those listed in Table 3.

GC–MS post-silylation analysis was employed to characterize *A. katsumadai* seed’s chemical profile, with emphasis on primary metabolites not detected using LC-MS, resulting in the identification of 48 metabolites that may contribute to its food or health effects. These metabolites were categorized into diarylheptanoids, organic acids, alcohols, amino acids, phenolic acids, flavonoids, sugars, fatty acids, mono-, and sesquiterpenes. In terms of quantity, the most abundant classes belonged to sugars 58.23%, arylheptanoids 10.05%, and flavonoids 8.88%. A list of identified compounds and their relative abundances is listed in Table 3, with the GC chromatogram displayed in Fig. 4.

**Table 3:** Quantification of silylated primary metabolites of AKS identified using GC–MS analysis. The values are expressed as average ± st. dev. (*n* = 3).

Peak #	Average Rt (min)	RI	Metabolite	mg/g ± st. dev.
*Organic acids*				
1	5.1877	1065.2	Lactic acid*	0.09 ± 0.01
2	5.8137	1104.1	Oxalic acid	0.17 ± 0.09
11	11.2393	1488.2	Malic acid*	0.05 ± 0.02
25	16.178	1931.6	Acrylic acid	0.45 ± 0.24
*Amino acids/nitrogenous compounds*				
3	6.2199	1129.3	Valine	0.07 ± 0.01
8	9.9593	1386.7	Unknown nitrogenous compound	0.14 ± 0.03
12	11.6319	1519.9	2-Pyrrolidone carboxylic acid	0.05 ± 0.02
*Alcohols*				
6	8.3749	1273	Glycerol*	0.17 ± 0.04
14	12.6006	1597.9	Carotol	0.02 ± 0.004
*Fatty acids*				
15	13.0561	1636.6	Dodecanoic acid	0.02 ± 0
29	17.1101	2026.4	Palmitic acid*	0.96 ± 0.22
30	18.6768	2196.7	Oleic acid*	0.67 ± 0.19
31	18.8891	2221.5	Stearic acid	0.11 ± 0.01
46	23.4719	2807.5	Lignoceric acid	0.07 ± 0.05
47	23.8187	2855.2	Docosenoic acid	0.27 ± 0.12
*Sugars*				
17	15.0067	1815.4	Fructose*	2.96 ± 1.36
18	15.0885	1823.5	Psicofuranose	4.44 ± 1.54
19	15.1687	1831.5	Fructose*	3.17 ± 1.81
20	15.4078	1855.2	Arabinose	0.35 ± 0.11
22	15.8748	1901.5	Fructose*	0.41 ± 0.26
23	15.9159	1905.6	Mannose	6.79 ± 3.73
24	16.0241	1916.4	Galactose	0.13 ± 0.03
27	16.7725	1990.6	Glucose*	10.07 ± 4.55
45	22.5106	2675.2	Sucrose*	23.81 ± 11.07
*Sugar alcohols*				
26	16.4052	1954.2	D-Mannitol	0.31 ± 0.12
*Sugar acids*				
28	17.0443	2019.3	D-Gluconic acid	0.28 ± 0.16
*Arylheptanoids/phenylbutanoids* ^#^				
4	6.9192	1172.8	1,7-Diphenyl-6(E)-hepten-3-ol	0.05 ± 0.03
32	20.5302	2416.4	5-Hydroxy-1,7-diphenyl-3-heptanone	0.03 ± 0.01
35	20.7356	2442.7	1-Phenyl-1-hepten-3-ol	0.22 ± 0.06
37	20.9073	2464.7	4-Phenyl-3-buten-2-ol	0.91 ± 0.33
38	20.9473	2469.8	1-Phenyl-1-hepten-3-ol	8.51 ± 2.43
39	21.2294	2505.9	(4E,6E)−1,7-Diphenyl-4,6-heptadien-3-one	0.33 ± 0.13
*Phenolic acids* ^#^				
5	7.9028	1239.2	Benzoic Acid	0.07 ± 0.01
13	11.8373	1536.4	Cinnamic acid	0.04 ± 0.02
43	21.9092	2593	Methyl mandelic acid	0.5 ± 0.08
*Monoterpenes/sesquiterpenes hydrocarbons* ^#^				
7	8.9997	1317.9	α-Terpineol	0.06 ± 0.02
9	10.8302	1455.3	Humulene	0.17 ± 0.02
16	14.7996	1795.3	Farnesol	2.32 ± 0.43
*Flavonoids/non-phenolic acids compounds* ^#^				
10	11.0659	1474.3	Phloroglucinol	0.14 ± 0.03
21	15.6344	1877.7	Catechin	0.35 ± 0.02
33	20.5891	2424	Cardamonin	1.03 ± 0.07
34	20.663	2433.4	Alizarin	0.07 ± 0.02
41	21.4623	2535.8	3,4-Dihydroxybenzophenone	1.87 ± 0.55
42	21.7067	2567	Cardamonin	5.43 ± 1.24
48	23.873	2862.7	(Epi)catechin	2.07 ± 0.41
*Unknowns* ^#^				
36	20.8003	2451	Unknown	1.24 ± 0.26
40	21.2941	2514.2	Unknown	5.77 ± 1.46
44	22.0305	2609.1	Unknown	3.46 ± 1.04

Asterisk denotes peaks confirmed by standard. ^#^Calculated as Relative abundance (%) ± St. dev.

#### Sugars/sugar alcohols/sugar acids

Sugars account for the seed’s nutritional value and taste, impacting its palatability^53^. In *A. katsumadai* seed, sugars amounting to the most dominant primary metabolites comprising 9 peaks. Sucrose (G45) was the main di-sugar, detected at 23.81 mg/g, constituting 26.51% of the total identified metabolites. In contrast, mono-sugars were detected but at much lower levels represented by fructose (G17, G19, G22), arabinose (G20), mannose (G23), and glucose (G27). Regarding sugar alcohols, only D-mannitol (G26) was detected at a low level, whereas D-gluconic acid (G28) was the only identified sugar acid, suggesting that di-sugars account the most for *A. katsumadai* seed calorie and taste.

#### Fatty acids

Fatty acids constitute an essential group of primary metabolites, represented by 6 distinct peaks observed at comparatively lower levels. The fatty acid composition of AKS was analyzed, revealing the presence of saturated and unsaturated fatty acids. Among the saturated fatty acids, palmitic acid (G29), a long-chain saturated fatty acid, was detected as the most abundant, constituting 0.96 mg/g. Other detected saturated fatty acids included dodecanoic acid (G15), stearic acid (G31), and lignoceric acid (G46). In addition, two monounsaturated fatty acids were identified: oleic acid (G30), which accounted for 0.67 mg/g and cis-13-docosenoic acid (G47), comprising 0.27 mg/g.

#### Organic acids

Organic acids play a crucial role in food products as natural preservatives, supporting digestion and enhancing protein utilization while also exhibiting antioxidant activity^11,18^. Acrylic acid (G25) was the major form accounting for 0.45 mg/g representing 1.61% of total identified metabolites, and likely to contribute for seed taste, followed by oxalic acid (G2), which was detected at 0.17 mg/g. Moreover, lactic acid (G1) and malic acid (G11) were found in trace amounts.

#### Alcohols

Alcohols were found at much lower levels, primarily represented by glycerol (G6), a simple triose compound with diverse pharmaceutical applications^54^. Additionally, a trace presence of carotol (G14), a sesquiterpene alcohol, was detected.

#### Amino acids/Nitrogenous compounds

In AKS, amino acids were detected at trace levels compared to other chemical classes represented by valine (G3) and 2-pyrrolidone carboxylic acid (G12) which were detected at 0.07 and 0.05 mg/g, respectively. Besides, a higher level of unknown nitrogenous compound (G8) was detected at 0.14 mg/g.

#### Arylheptanoids

A total of 6 arylheptanoids were detected in *A. katsumadai* seeds including three diarylheptanoids (G4, G32, G39), two phenylheptanoids (G35), and 1-phenyl-1-hepten-3-ol (G38), which exhibited the highest level at 8.51% of the total identified metabolites and 84.13% of the identified arylheptanoids, in addition to a peak of an arylbutanoid compound (G37).

#### Flavonoids and non-phenolic acid compounds

Flavonoids constituted a major class represented by two catechins peaks (G21, G48) and two peaks (G33, G42) of cardamonin, the predominant flavonoid compound accounting for 6.46% of the total identified metabolites. It plays a key role in imparting the characteristic strong aroma of AKS, giving them a scent like that of cardamom^5,21^. Additionally, alizarin (G34), a dihydroxyanthraquinone metabolite, was detected at a low abundance percentage of 0.07%. Other phenolic derivatives, such as phloroglucinol (G10) and 3,4-dihydroxybenzophenone (G41), were also detected at levels of 0.14% and 1.87%, respectively.

#### Phenolic acids

Compared to other metabolite classes, phenolic acids were also identified in AKS at relatively lower levels. Three phenolic acids were detected, including benzoic acid (G5) and cinnamic acid (G13), with trace levels. Notably, methyl mandelic acid (G43) was the predominant phenolic acid, comprising 0.5% of the total identified metabolites.

## Conclusion

The current study presented a comprehensive investigation of AKS metabolome, targeting primary and secondary metabolites as well as aroma constituents to decode for seed health-promoting properties and culinary value. UHPLC-ESI-MS- MS/MS-based GNPS networking allowed for the annotation of 82 secondary metabolites across diverse chemical classes. Such extensive profiling led to the detection of two newly annotated catechin-guibourtinidol derivatives and deoxy phloretin-3’-C-hexoside, identified for the first time in the *Alpinia* genus. As well, SPME headspace aroma profiling identified a total of 30 volatiles belonging to various classes, with the abundance of monoterpenes and sesquiterpenes accounting for its discrete aroma. Results of GC–MS post-silylation analysis resulting in the identification of 48 metabolites, revealing the predominance of sugars, arylheptanoids, and flavonoids. Among these, sucrose emerged as the most abundant sugar, while cardamonin was identified as the predominant flavonoid. These components significantly underpin the seed’s dual role in culinary and medicinal applications: sucrose imparts a delicate sweetness, while cardamonin contributes to its distinctive aromatic flavor profile alongside certain potent pharmacological actions, such as anti-inflammatory and anticancer activities. Future investigations employing other broader analytical techniques targeting minerals, vitamins in AKS are recommended to identify optimal resources and further elucidate their health-promoting effects.

## Materials and methods

### Plant material

*Alpinia katsumadai* seeds were purchased from local store in Hangzhou China and authenticated by Dr. Zuying Zhang, Zhejiang A&F University, Hangzhou, Zhejiang, China. A voucher specimen (No. 10-9-25-F) was placed at the herbarium of the Faculty of Pharmacy, Cairo University.

### Chemicals

Formic acid and acetonitrile (HPLC grade) were provided by Baker (Deventer, The Netherlands). All other solvents, standards, and chemicals were obtained from Sigma Aldrich (St Louis, MO, USA). For SPME sampling, Special fibers of polydimethylsiloxane or divinylbenzene/carboxen/polydimethylsiloxane (DVB/CAR/PDMS, 50 μm/30 µm of 1 cm in length) were obtained from Supelco (Bellefonte, PA, USA). All solvents, standards and chemicals were obtained from Sigma Aldrich (St. Louis, MO, USA).

### UHPLC-ESI-QTOF-MS/MS analysis and GNPS feature-based molecular MS/MS network

Finely ground AKS (10 mg) was subjected to extraction with 2 mL of 70% MeOH, containing 10 µg mL^−1^ umbelliferone (internal standard) and sonication for 20 min with intermittent shaking, then centrifugation at 12 000 × g for 10 min. Then the extract was filtered through a 0.22-µm filter and subjected to solid-phase extraction using a C18 cartridge (SepPack, Waters, Milford, MA, USA). 2 µL of the extract were injected on an HSS T3 column (100 × 1.0 mm, particle size 1.8 μm; Waters, Milford, MA, USA) installed on an ACQUITY UPLC system (Waters) equipped with a 6540 Agilent Ultra-High-Definition (UHD) Accurate-Mass Q-TOF-LC–MS (Palo Alto, CA, USA) coupled to an ESI interface, operated in positive or negative ion mode under the exact conditions as previously described^55^. Duplicate runs per sample were performed.Metabolites were characterized by their exact masses, MS/MS fragmentation in both positive and negative ionization modes, retention time, and comparisons to the Natural Products database and reference literature. Using the FBMN workflow on GNPS2, a molecular networking was created. The resulting aligned list of features was exported in an mgf file besides their feature quantification table in csv format. The values of feature quantification table were uploaded onto the FBMN page of GNPS2. Edges of the MN were filtered to have a cosine score above 0.65 and more than 4 matched peaks between the connected nodes. The edges between two nodes were kept in the network. The MNs were visualized using Cytoscape 3.9.1. For molecular networking of AKS in positive mode (https://gnps2.org/status? task=ae9207b6aa644d48ac4e7f9515cb6f90 ), and in negative mode (https://gnps2.org/status? task=bc6a6741185343c0a06afb68aaf4828c ), the precursor ion mass tolerance was set to 0.02 Da and the MS/MS fragment ion tolerance to 0.02 Da.

### SPME GC–MS volatiles analysis

100 mg of freeze-dried seed powder was loaded into 1.5 mL SPME vials spiked with 10 µg (*Z*)−3-hexenyl acetate. After fiber insertion above, samples were equilibrated at 50 °C for 30 min. Volatile analysis was performed by HS-SPME/GC–MS using an Agilent 5977B GC/MSD with a DB-5 column (30 m × 0.25 mm × 0.25 μm film thickness; Supelco) and a quadrupole mass spectrometer. The injector and interface temperatures were maintained at 220 °C. The oven temperature program was initiated at 40 °C for 3 min, ramped to 180 °C at 12 °C/min (held 5 min), then to 240 °C at 40 °C/min (held 5 min). Helium carrier gas flowed at 0.9 mL/min. Post-analysis, fibers were reconditioned at 220 °C for 2 min. Triplicate runs per sample were performed, included interspersed blanks, with EI-MS operating at 70 eV (scan range: m/z 40–500)^56^. Volatiles were quantified relative to the amount of recovered hexenyl acetate as an internal standard.

### GC–MS analysis of silylated primary metabolites

Freeze-dried seed powder (100 mg) was extracted with 5 mL 100% methanol with sonication (30 min, frequent vortex shaking). Triplicate extracts per accession were processed. Aliquots (100 µL) were evaporated under a nitrogen gas stream, then derivatized with 150 µL N-methyl- N-(trimethylsilyl)-trifluoroacetamide (MSTFA, Sigma, St. Louis, MO, USA)/anhydrous pyridine (1:1) at 60 °C for 45 min prior to GC–MS analysis. Silylated derivatives were separated on a Rtx-5MS column (30 m × 0.25 mm, 0.25 μm). Quantitative analysis of primary metabolites followed^54^. Soluble sugars, amino acids, organic acids and fatty acids were quantified using standard curves of glucose, glycine, citric and stearic acid standards (Four serial dilutions of each, from 10 to 600 µg/mL; R² ≈ 0.99). Results were expressed as mg/g dry weight^56^. GC–MS data were processed using AMDIS software (www.amdis.net) for peak deconvolution before mass spectral matching. Identification of both volatile and silylated components was based on calculated KI using alkane standard C8-C40, mass matching to NIST database and standards whenever available as the exact protocol detailed in^57^. Peak abundance was obtained using MS-DIAL software following default parameters for GC/MS^53^.

## Supplementary Information

Below is the link to the electronic supplementary material.

Supplementary Material 1

## Data Availability

The authors declare that the data supporting the findings of this study are available within the paper and its supplementary information file.
