# Supplementary material for: Comprehensive metabolites characterization of Alpinia katsumadai seeds via a multiplex approach of UHPLC–MS/MS and GC–MS techniques
Source: Sci Rep. 2025 Dec 9;15:43390. doi: 10.1038/s41598-025-29831-4 (PMC12689793; doi:10.1038/s41598-025-29831-4)
Supplement: Supplementary file 1 — Supplementary Material 1 [file 41598_2025_29831_MOESM1_ESM.docx]

**Comprehensive metabolites characterization of *Alpinia katsumadai* seeds *via* a multiplex approach of UHPLC-MS/MS and GC-MS techniques**

**Nermeen B. Ali^1*^, Mai E. Hussein^1^, Mohamed A. Farag^1^****

*^1^ Pharmacognosy Department, Faculty of Pharmacy, Cairo University, Cairo, Egypt*

***Corresponding author**:

**E-mail address:** Nermeen.ali@pharma.cu.edu.eg

**Co-corresponding author: mohamed.farag@pharma.cu.edu.eg

**Postal address:** Department of Pharmacognosy, Faculty of Pharmacy, Cairo University, El-Kasr El-Aini St, 11562 Cairo, Egypt.


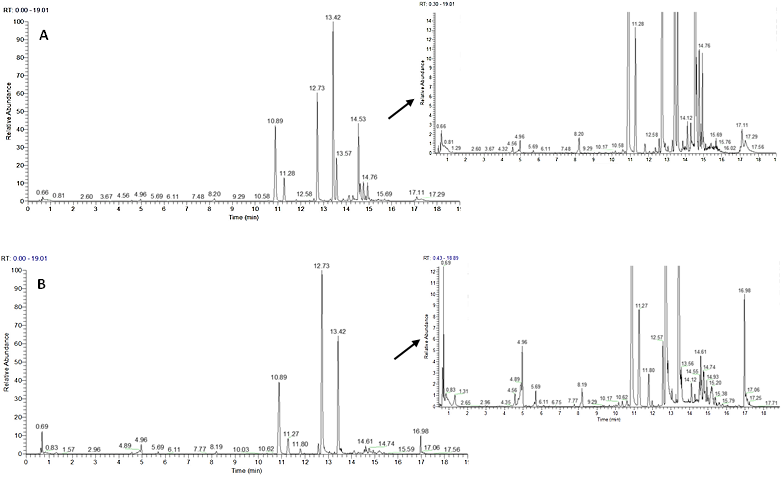


**Fig. S1**. UHPLC-MS/MS chromatograms of methanolic extract of AKS in positive mode (A) and in negative mode (B).


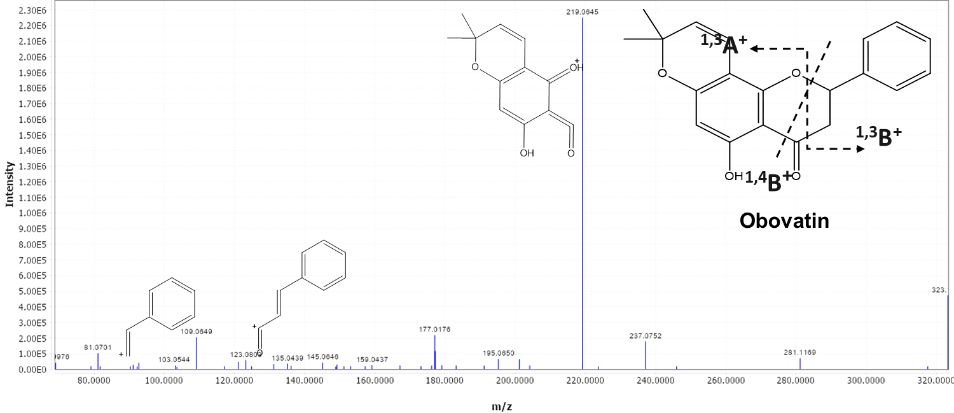

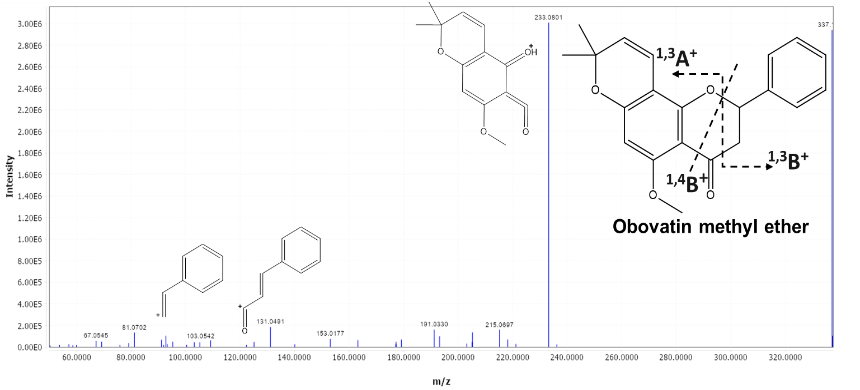

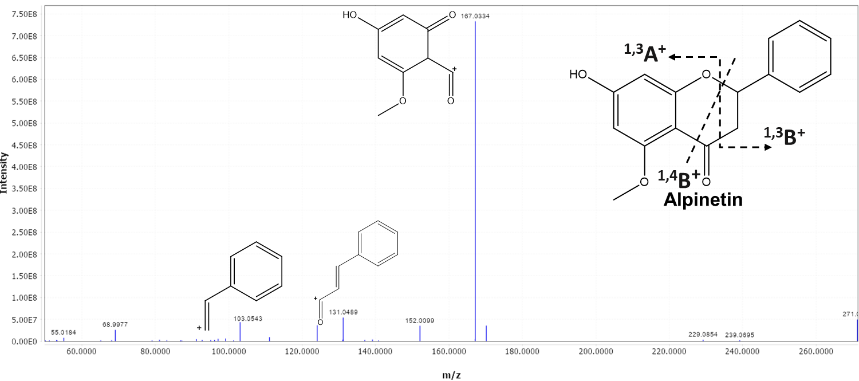

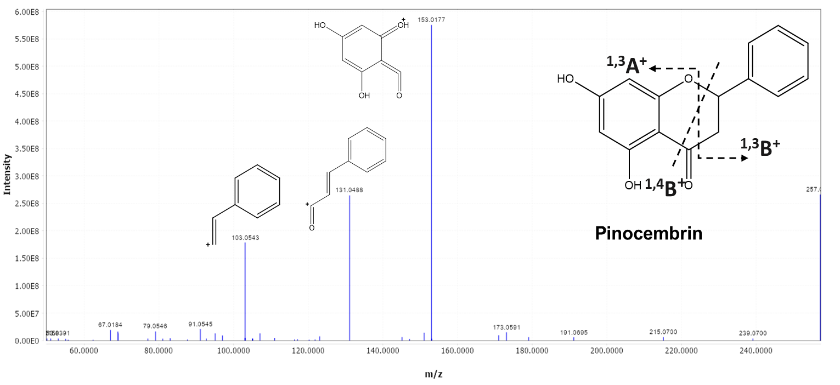

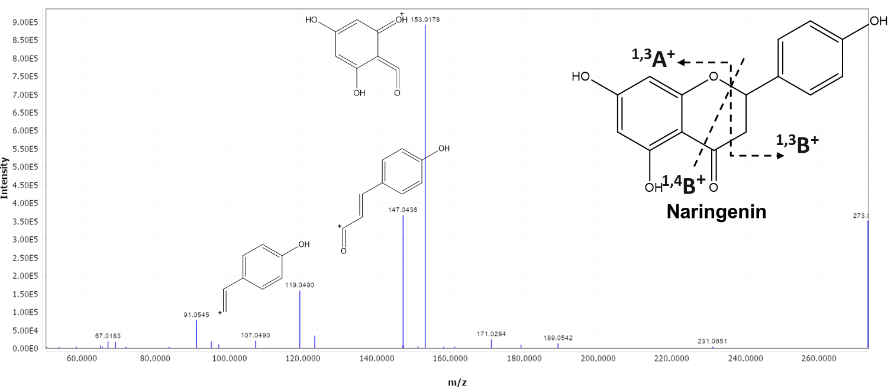


**E**

**D**

**C**

**B**

**A**

**Fig. S2**. MS/MS fragmentation of naringenin (L34, *m/z* 273.0750, R_t_:10.37) (A); pinocembrin (L36, *m/z* 257.0800, R_t_:12.73) (B); alpinetin (L38, *m/z* 271.0956, R_t_:13.42) (C), obovatin methyl ether (L39, *m/z* 337.1423, R_t_:14.12) (D), obovatin (L40, *m/z* 323.1268, R_t_:14.40) (E), respectively.


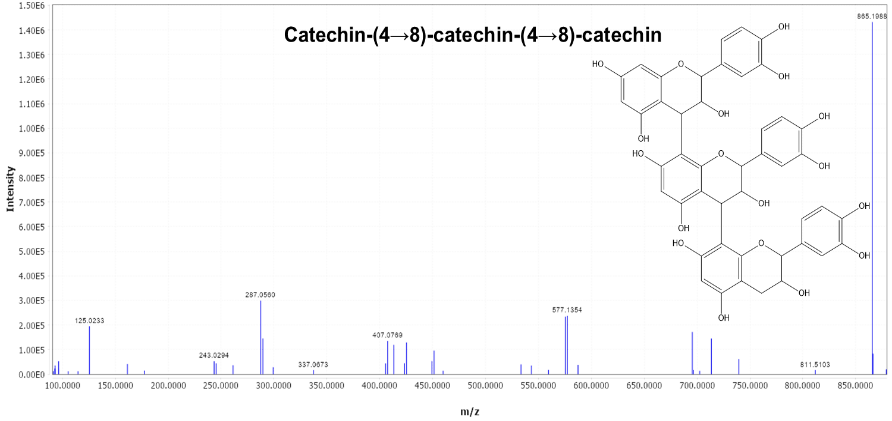

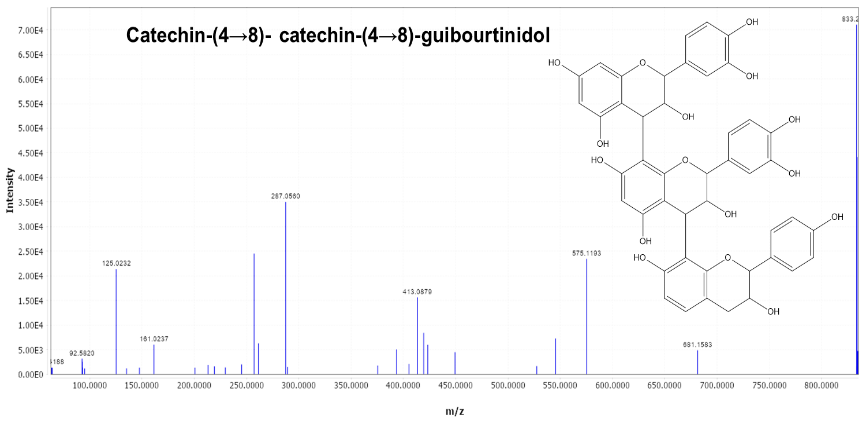

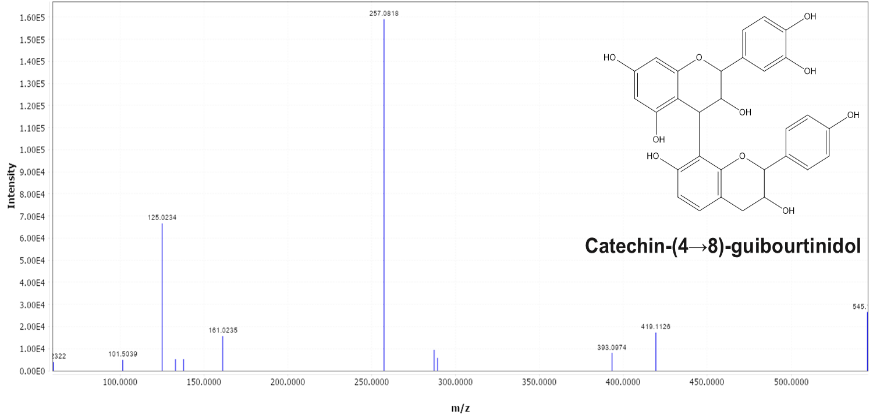

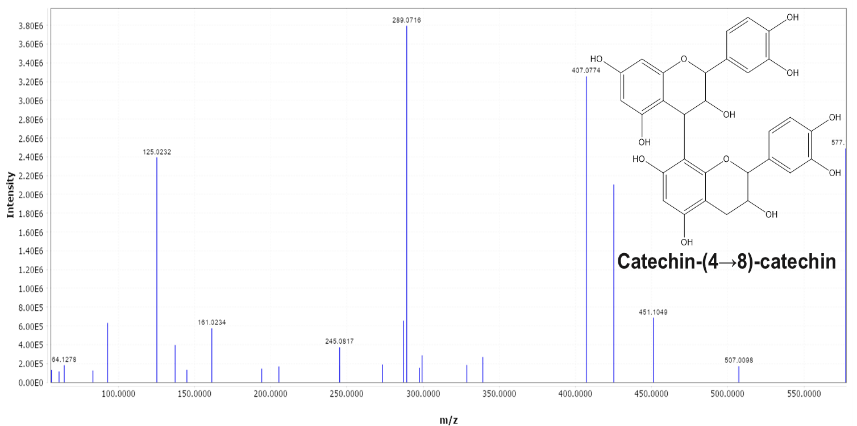


**D**

**C**

**B**

**A**

**Fig. S3**. MS/MS fragmentation of procyanidins; catechin-(4→8)-catechin (L45, *m/z* 577.1349, R_t_:4.56) (A); catechin-(4→8)-guibourtinidol (L50, *m/z* 545.1450, R_t_:11.56) (B); Catechin-(4→8)- catechin-(4→8)-catechin (L47, *m/z* 865.1982, R_t_:5.00) (C); catechin-(4→8)-catechin-(4→8)-guibourtinidol (L49, *m/z* 833.2090, R_t_:10.98) (D) , respectively.


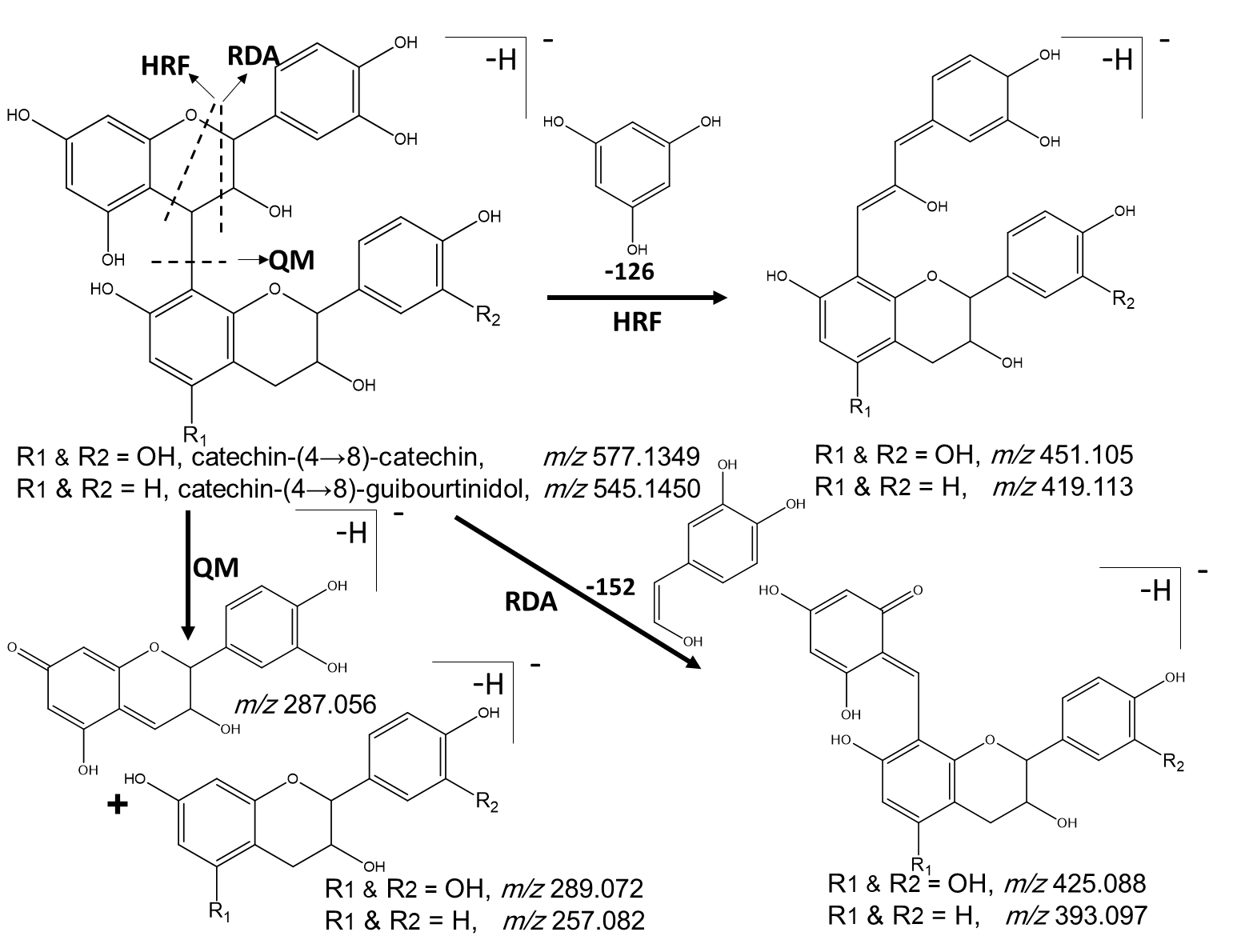

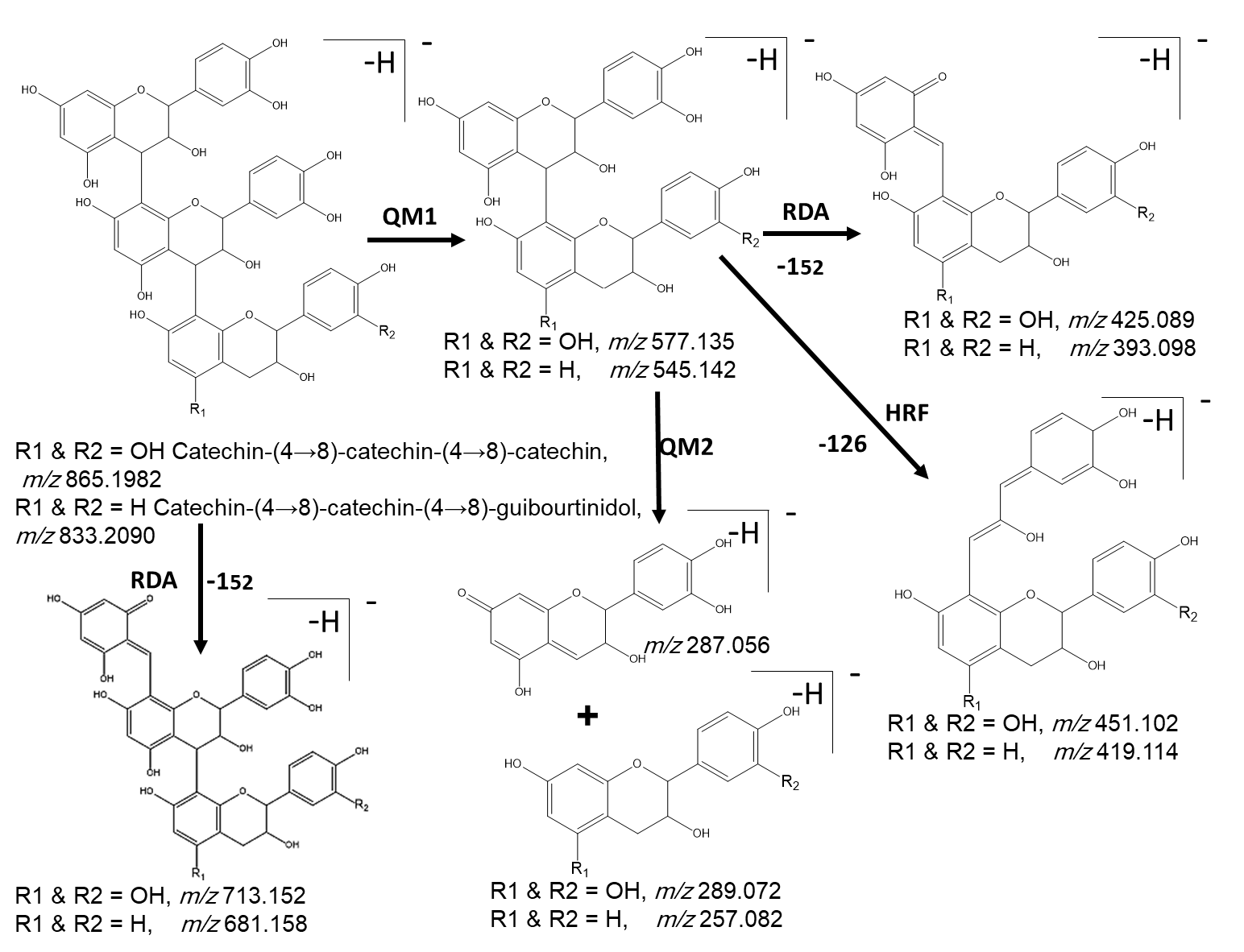


**A**

**B**

**a**

**b**

**Fig. S4**. Fragmentation pathway and the MS/MS of B-type procyanidins dimer (A) and B-type procyanidins trimer (B).


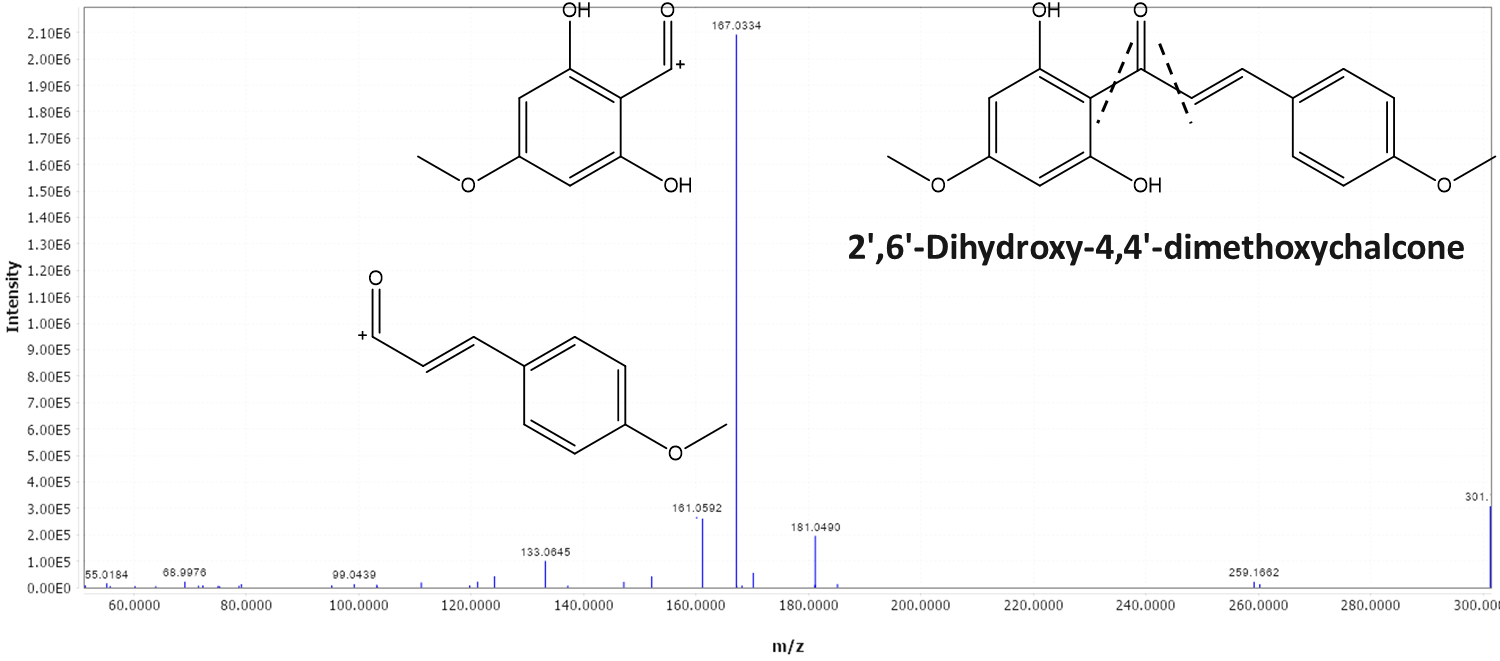

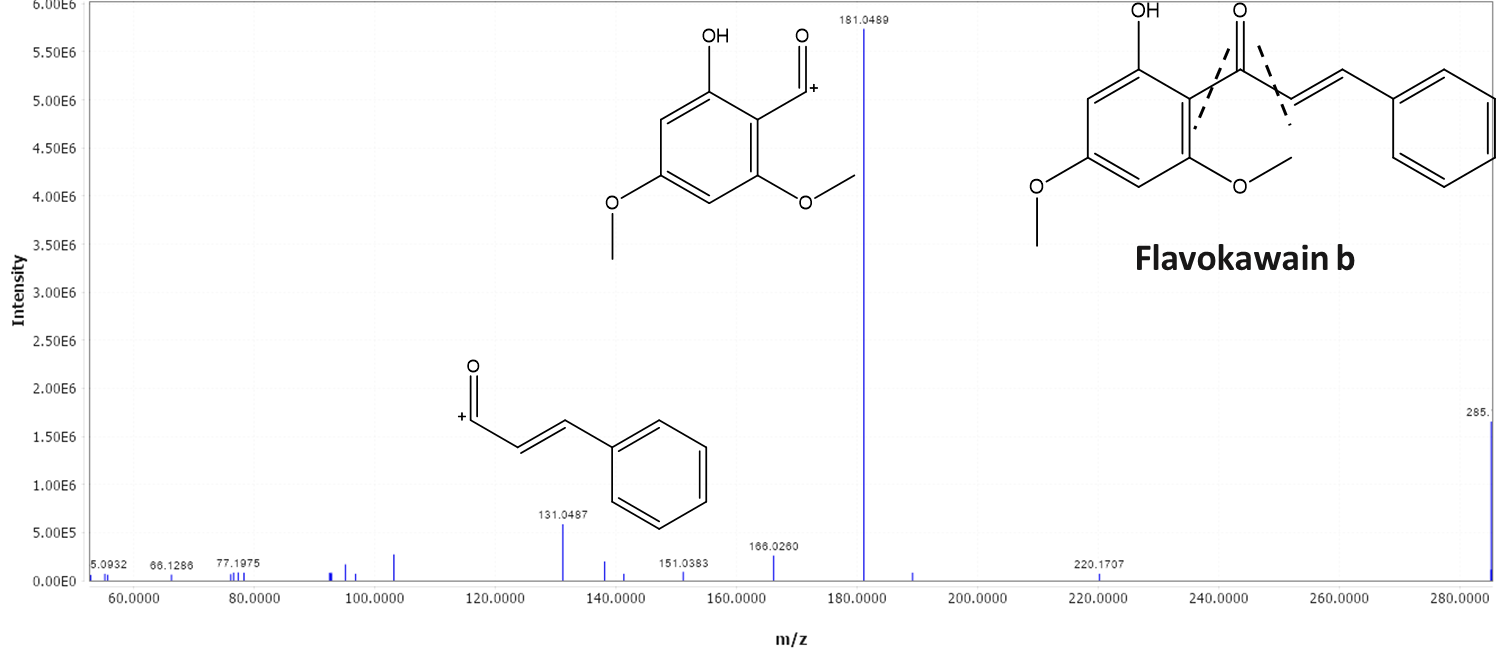


**A**

**B**

**Fig. S5**. MS/MS fragmentation of flavokawain b (L59, *m/z* 285.1112, R_t_:12.90) (A) and 2',6'-dihydroxy-4,4'-dimethoxychalcone (L61, *m/z* 301.1061, R_t_:13.32) (B), respectively.

**
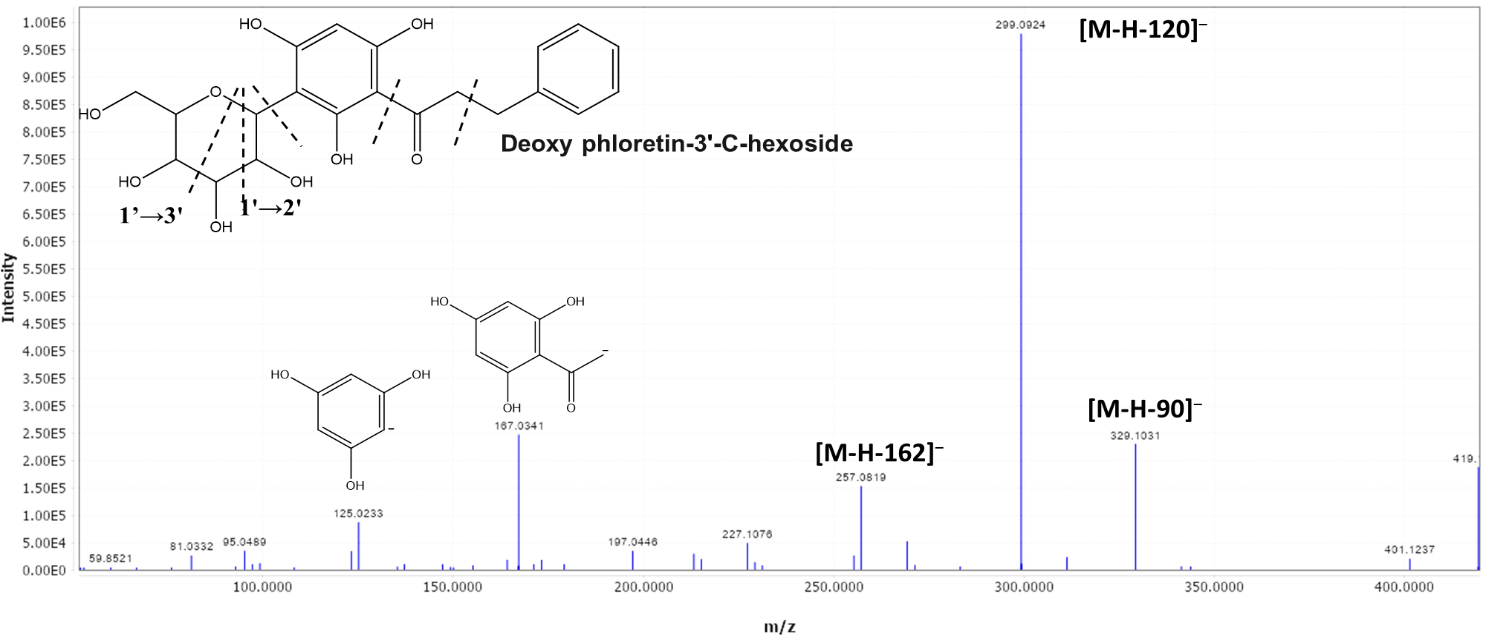
F****ig. S6**. MS/MS fragmentation of aspalathin (L51, *m/z* 451.1245, R_t_:3.43) (A); nothofagin (L53, *m/z* 435.1294, R_t_:7.45) (B) and deoxy phloretin-3'-C-hexoside (L55, *m/z* 419.1346, R_t_:9.80) (C), respectively.

**C**


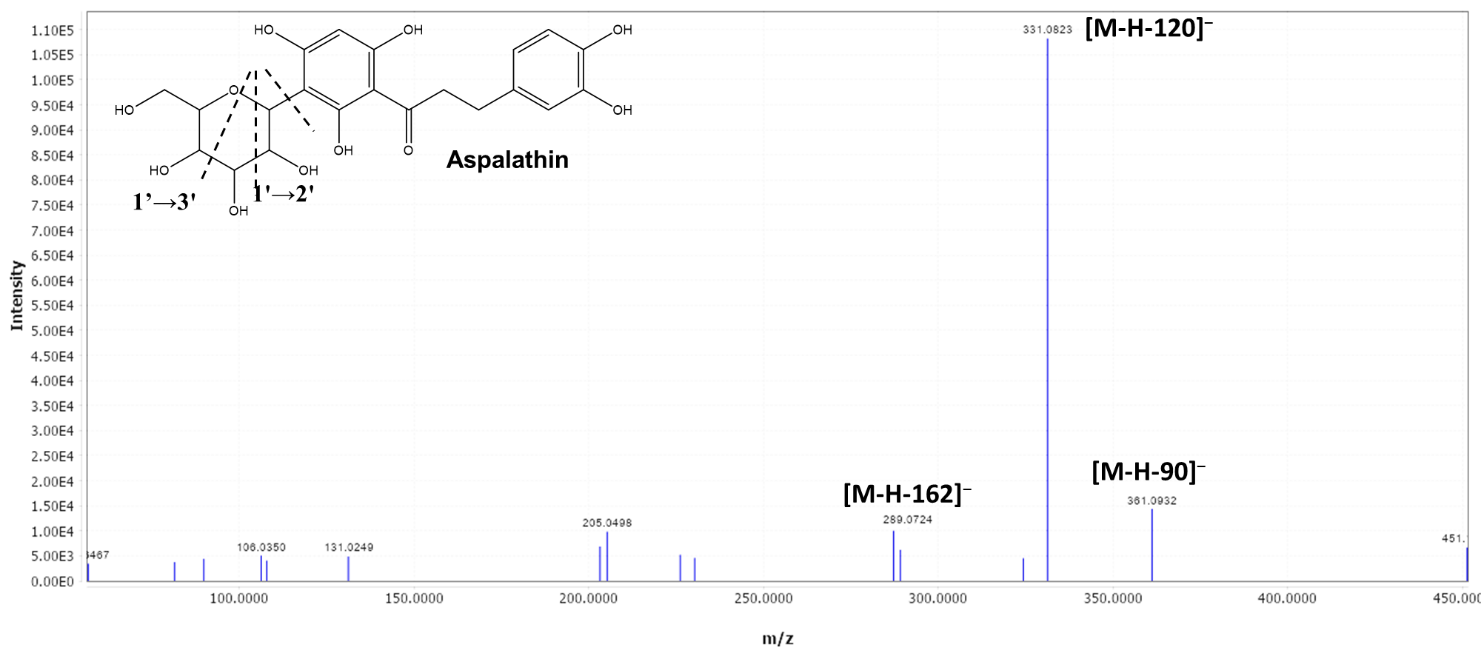

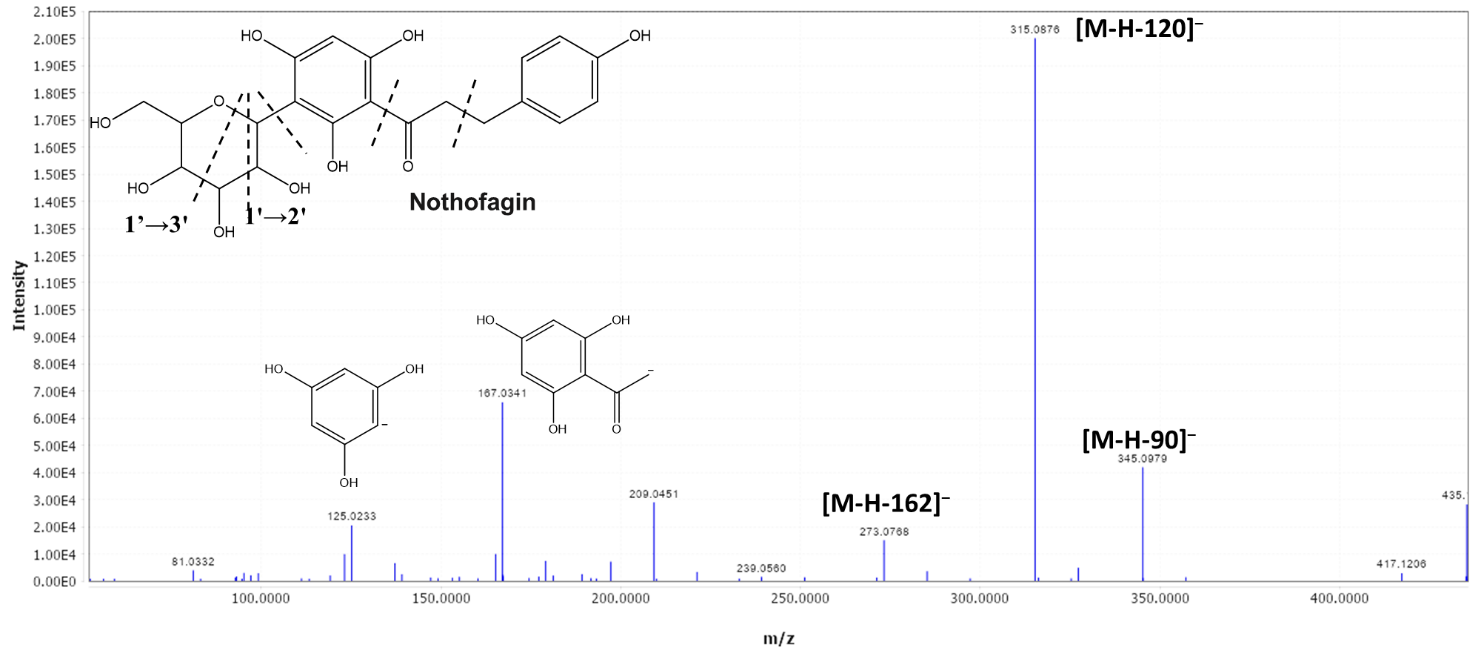


**B**

**A**

**F****ig. S7**. MS/MS fragmentation of phloretin-3',5'-di-C-hexoside (L52, *m/z* 597.1825, R_t_:7.03) (A); deoxy phloretin-3',5'-di-C-hexoside (L54, *m/z* 581.1873, R_t_:8.82) (B) and 2ʹ,4ʹ,6ʹ-trihydroxy-acetophenone-3ʹ,5ʹ-di-C-hexoside (L78, *m/z* 491.1401, R_t_:4.72) (C), respectively.


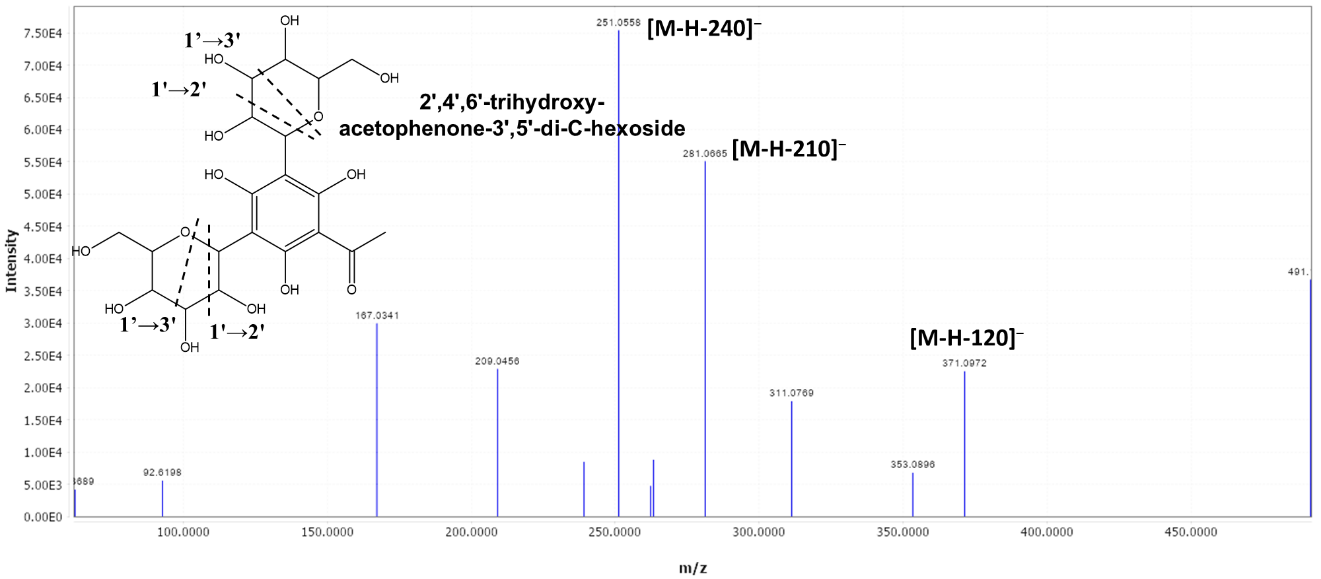

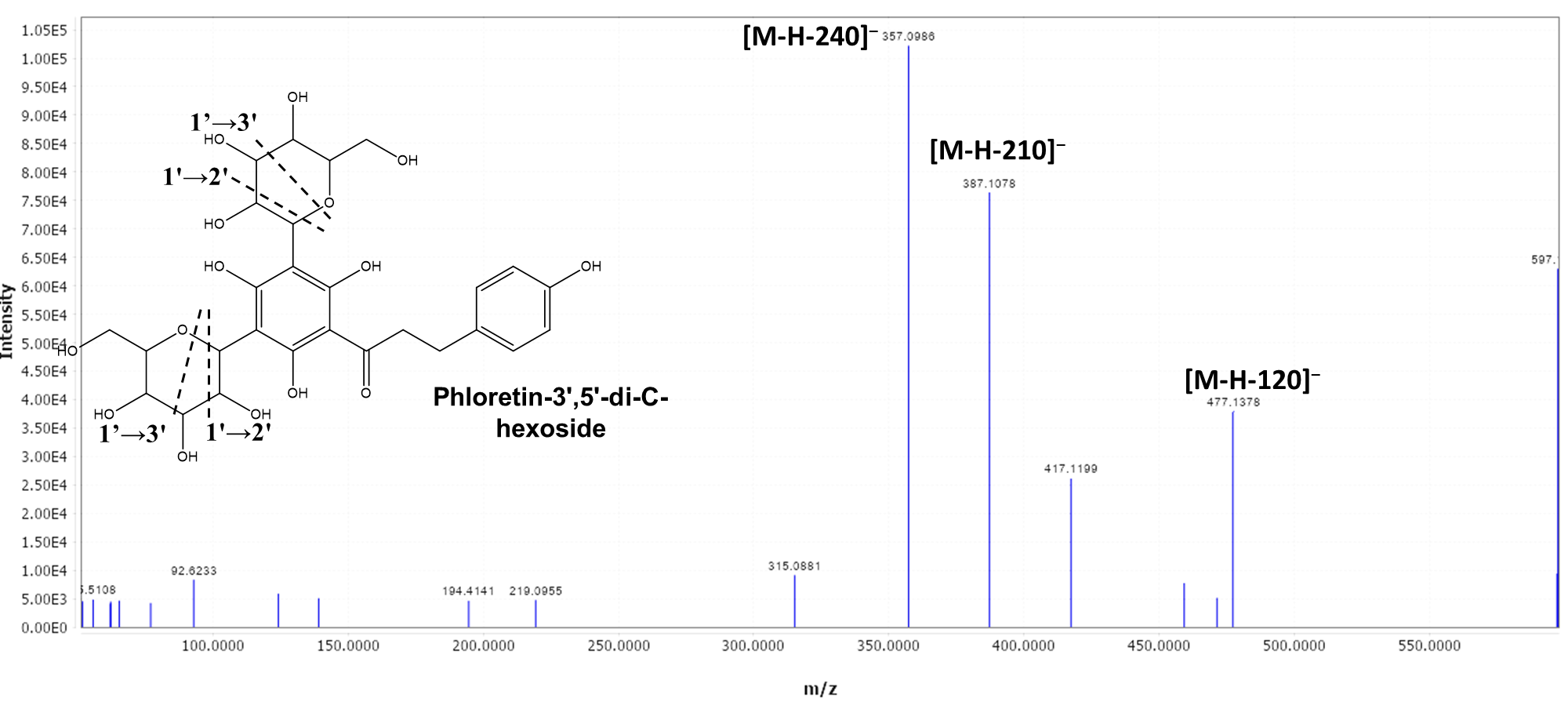

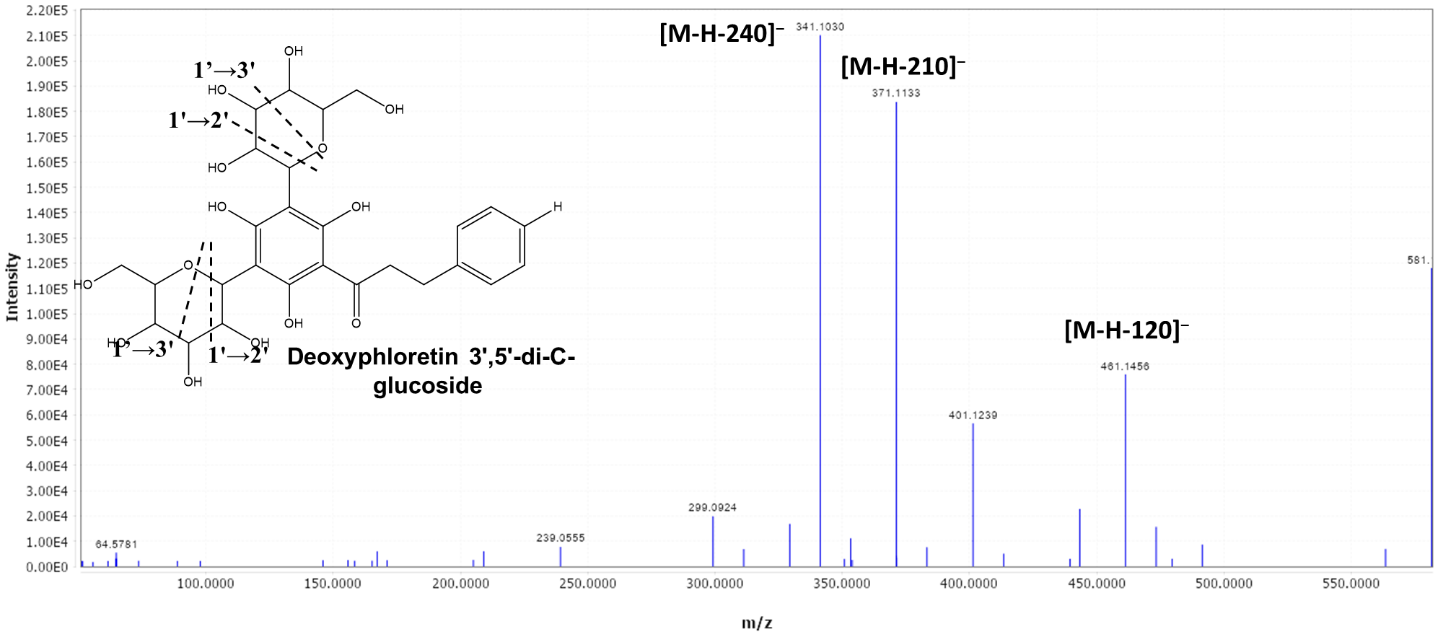


**C**

**B**

**A**

**A**


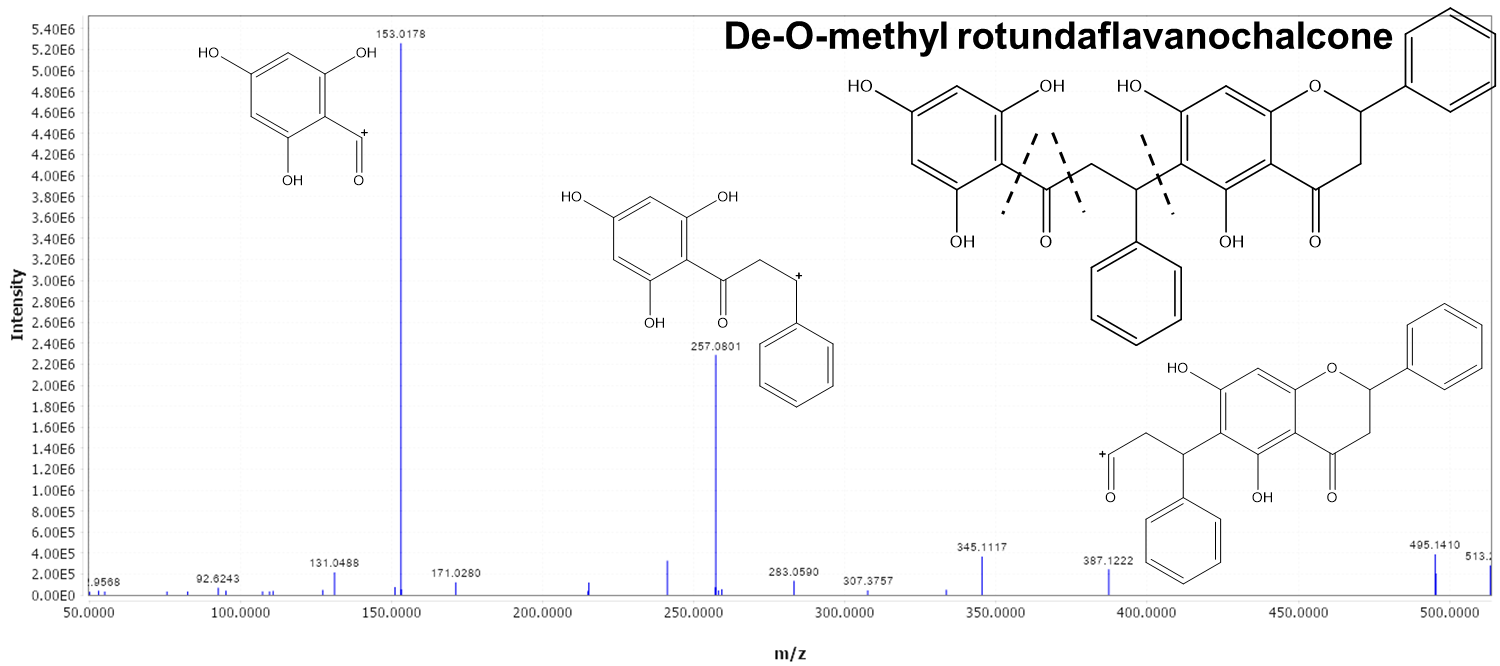

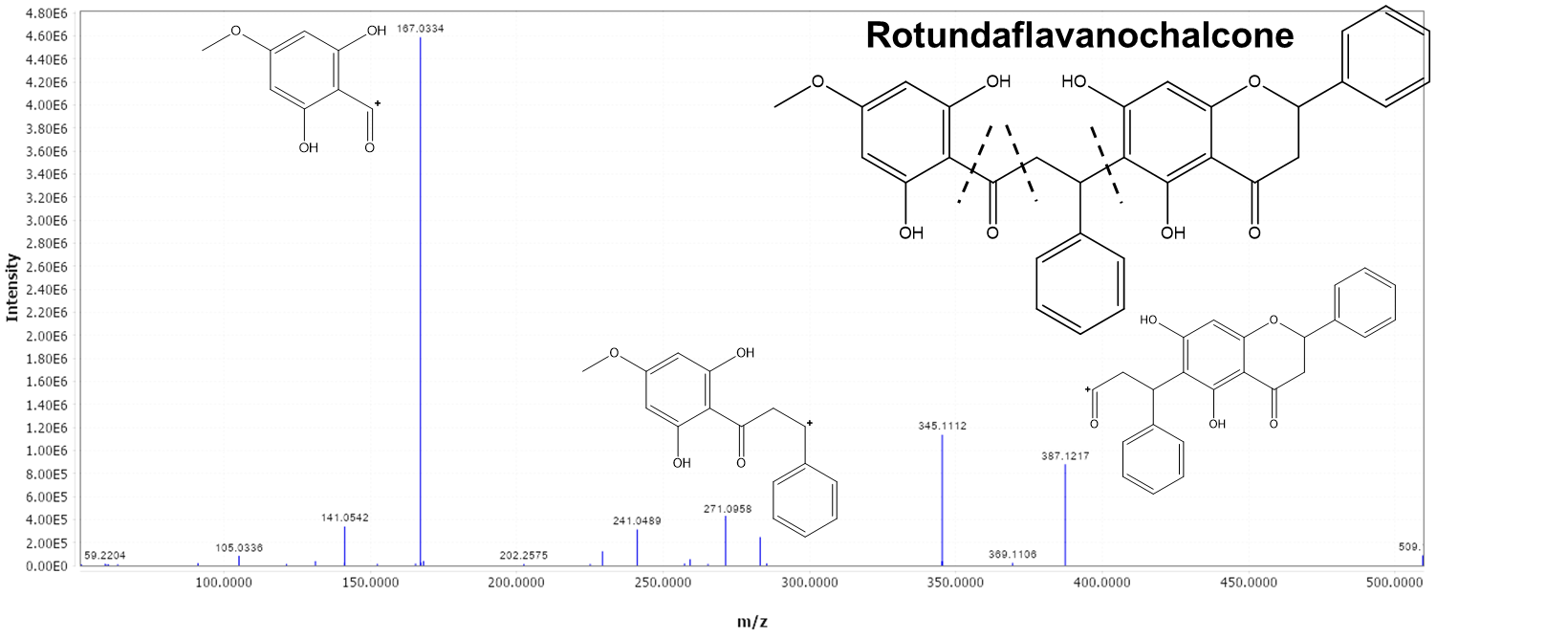


**B**

**Fig. S8**. MS/MS fragmentation of de-*O*-methyl rotundaflavanochalcone (L62, *m/z* 513.1532, R_t_:13.41) (A); rotundaflavanochalcone (L65, *m/z* 527.1689, R_t_:13.95) (B), respectively.


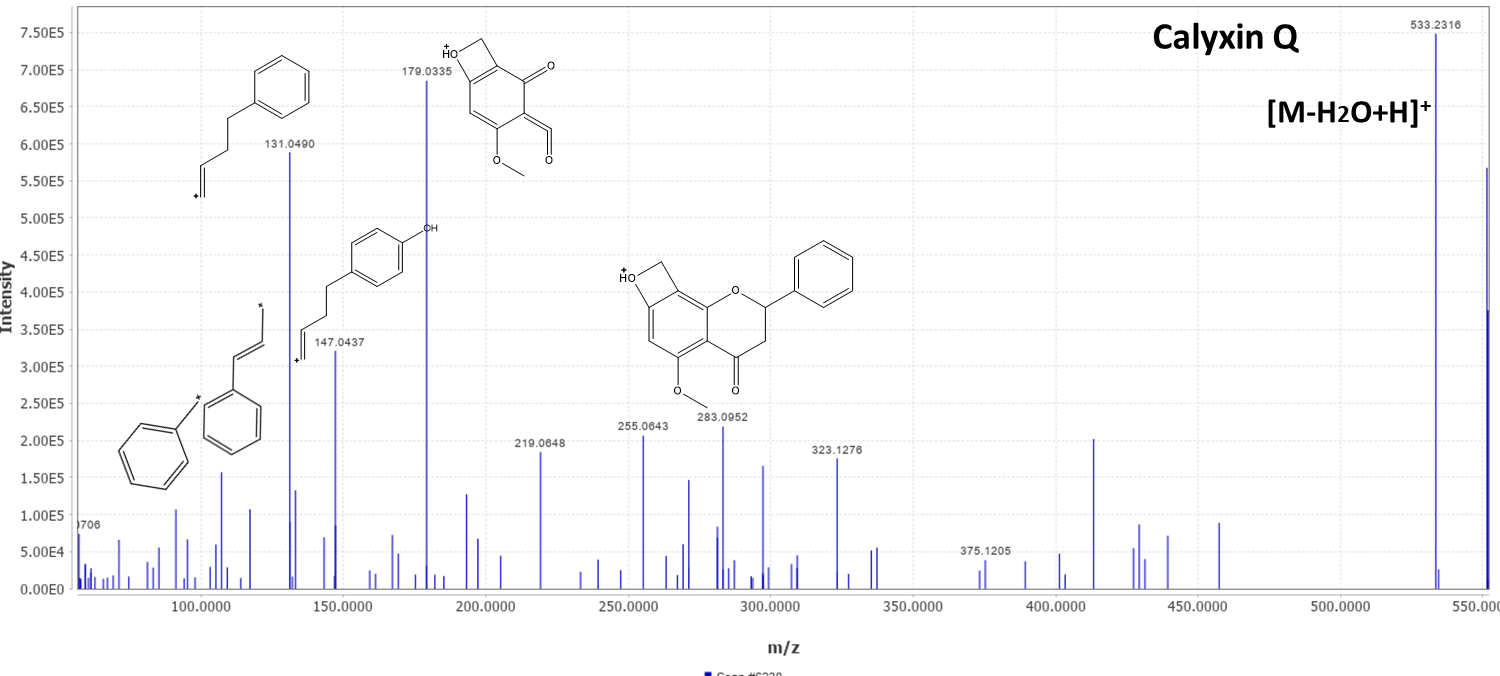
 **Fig. S9**. MS/MS fragmentation of calyxin N / O (L66, *m/z* 535.2467, R_t_:14.05) (A); calyxin Q (L67, *m/z* 551.2415, R_t_:14.24) (B), calyxin P (L68, *m/z* 521.2312, R_t_: 14.65) (C), respectively.

**B**

**C**


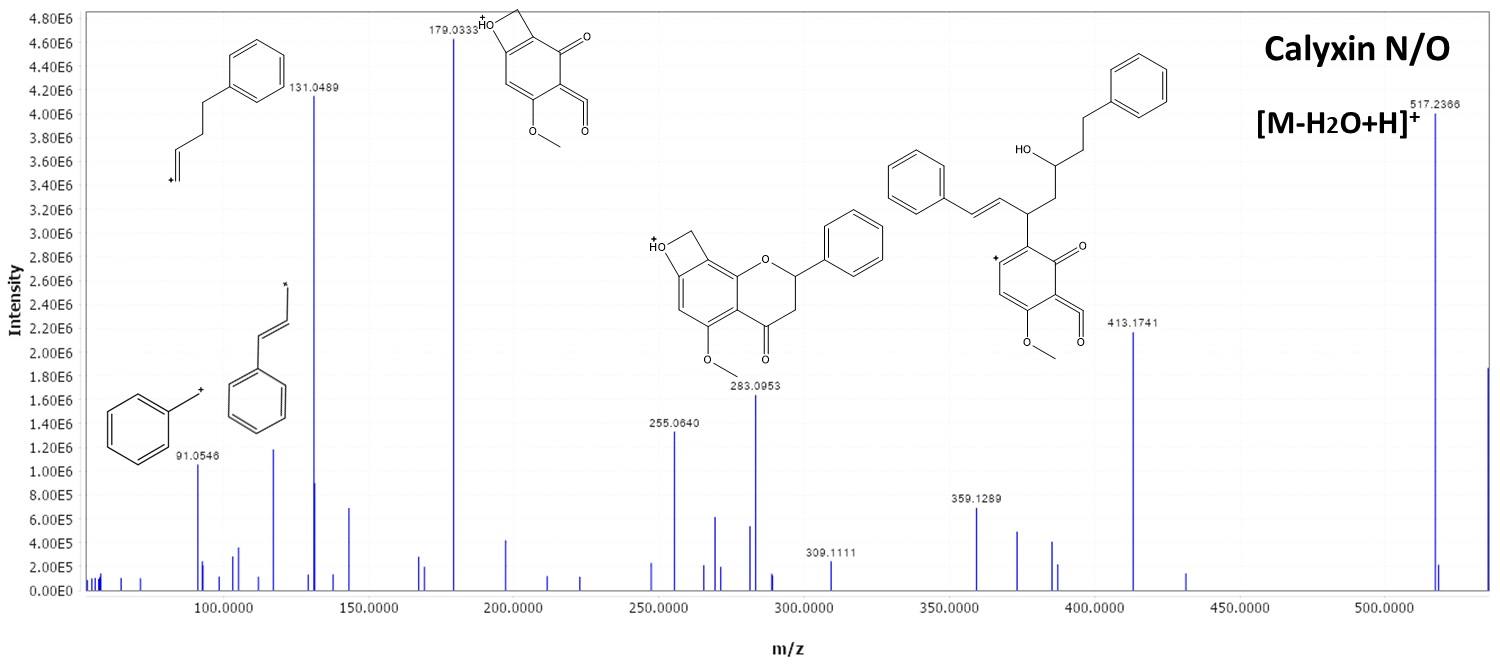

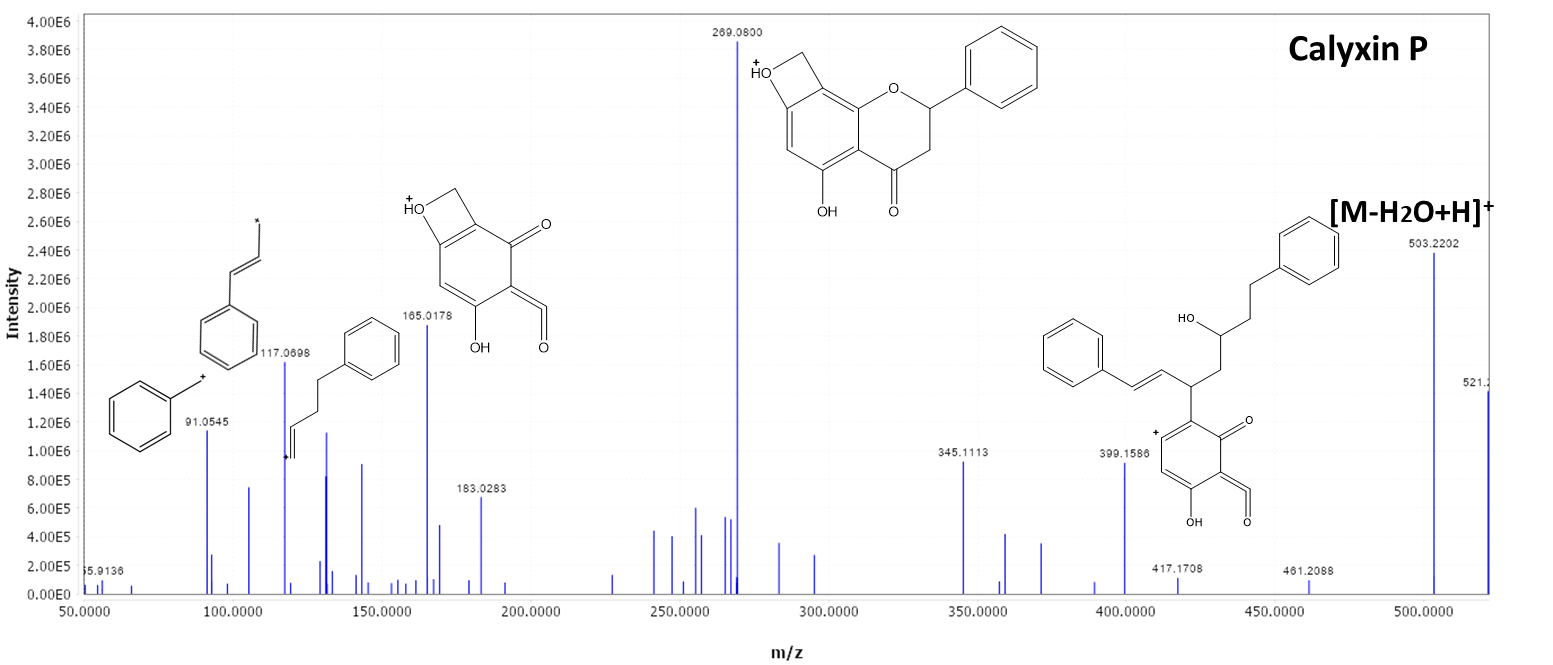


**A**


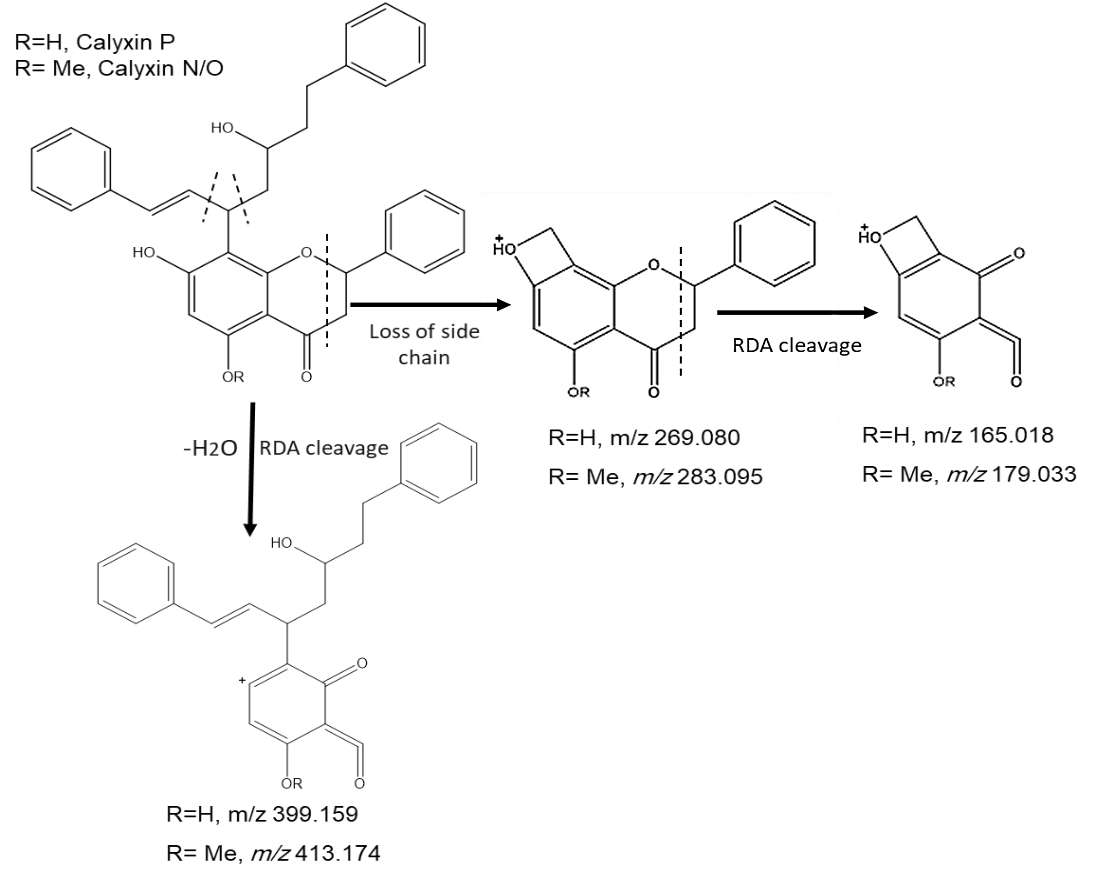


**A**

**B**


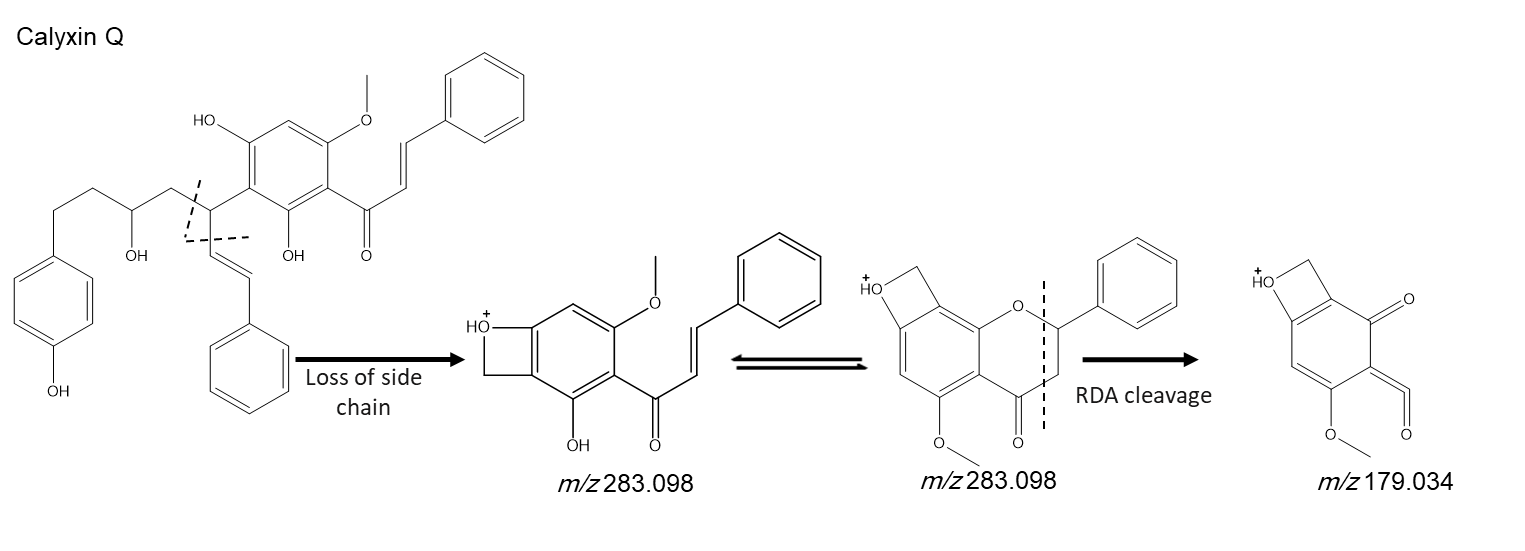


**Fig. S10.** Proposed mass spectrometric fragmentation pathways of calyxin N /O &P (A) and calyxin Q (B).

**A**


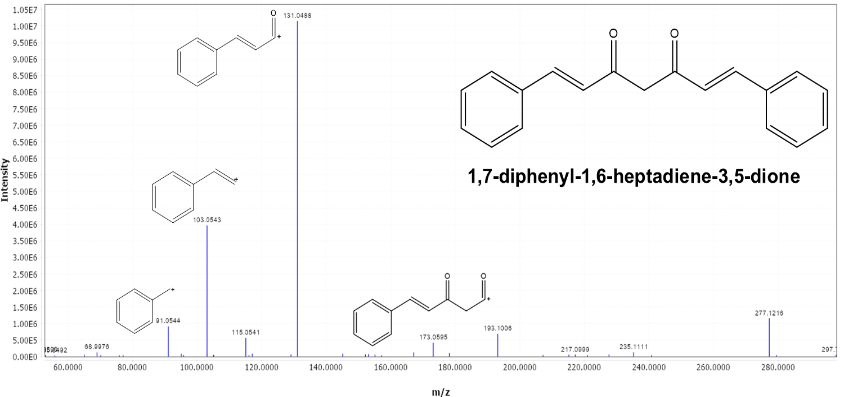

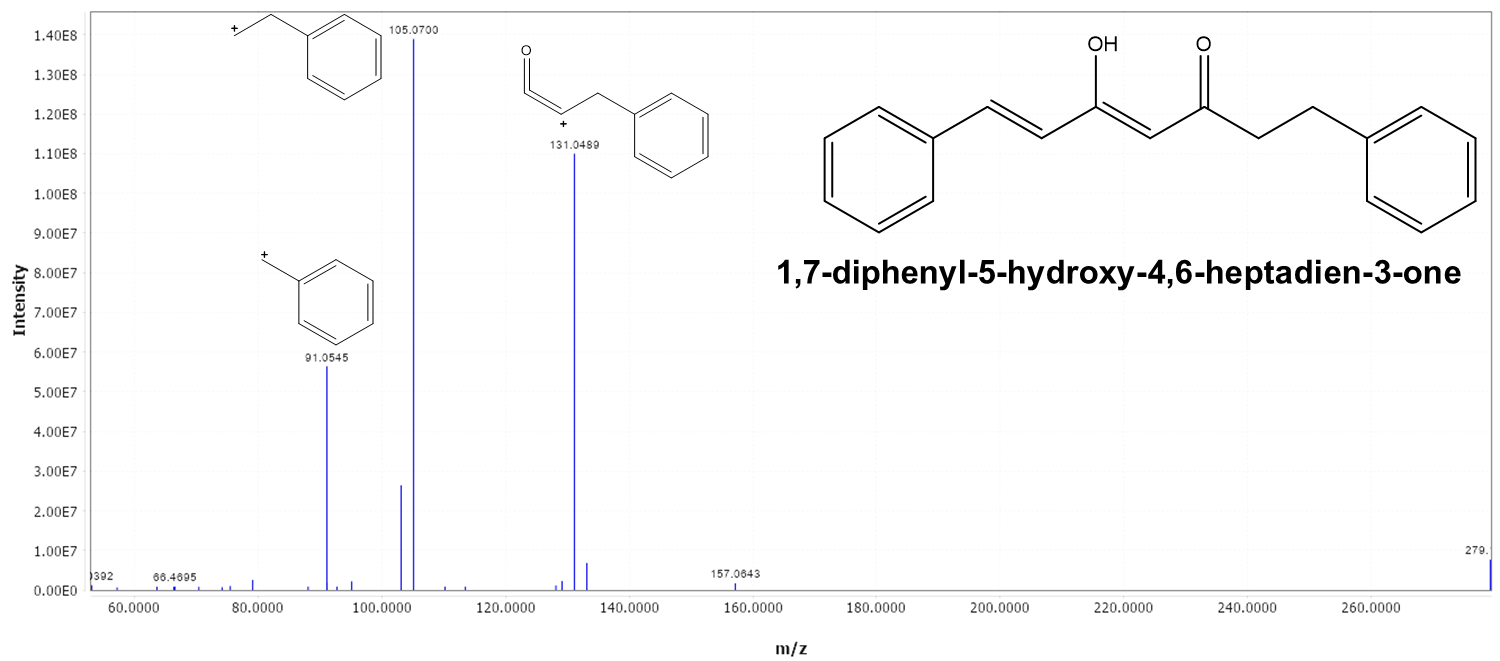

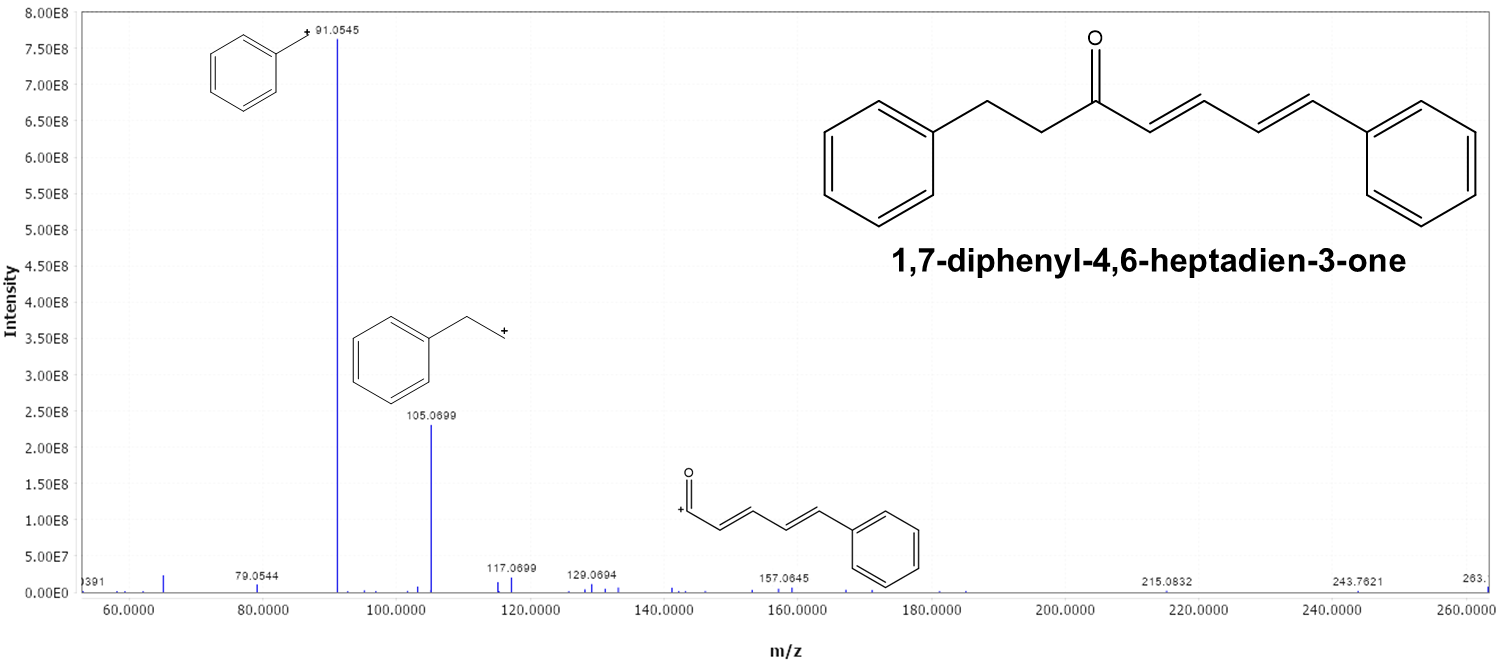

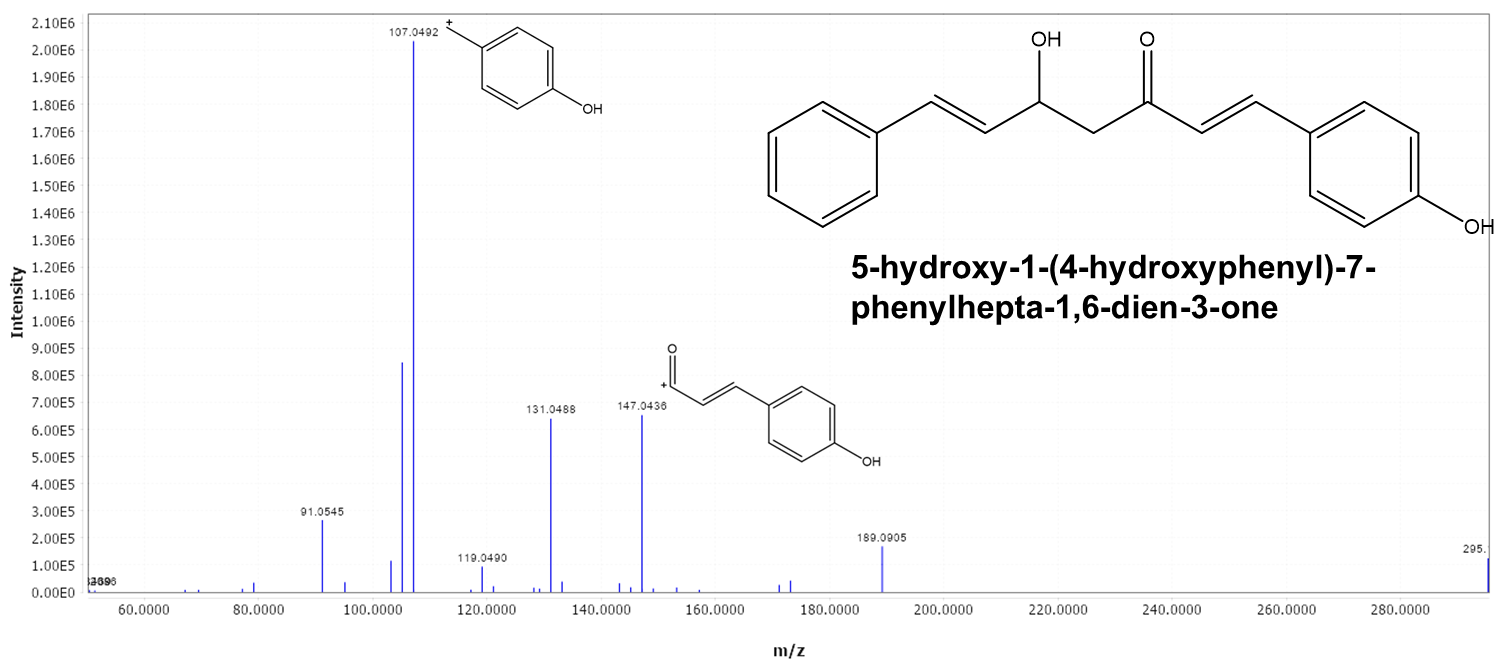

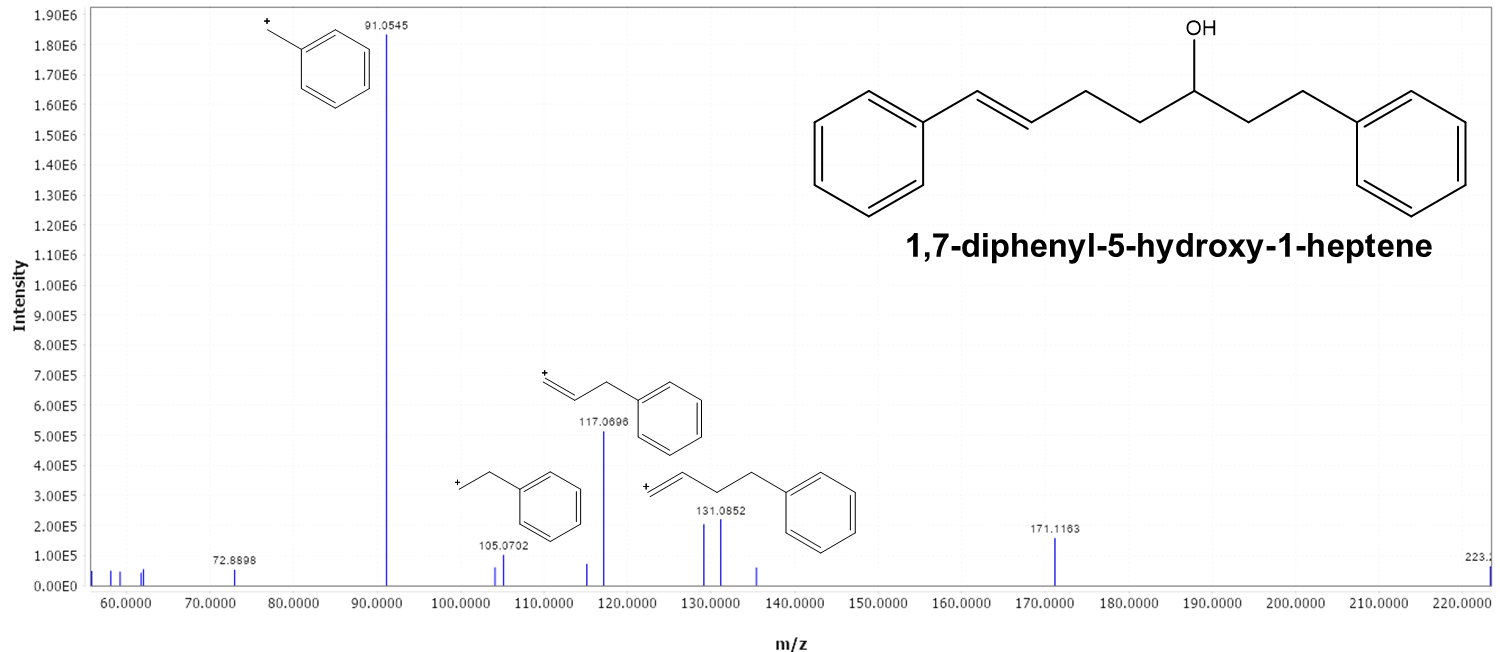


**E**

**B**

**D**

**C**

**Fig. S11**. MS/MS fragmentation of 1,7-diphenyl-5-hydroxy-1-heptene (L69, *m/z* 267.1734, R_t_:13.56) (A); 5-hydroxy-1-(4-hydroxyphenyl)-7-phenylhepta-1,6-dien-3-one (L70, *m/z* 295.1320, R_t_:13.95) (B), 1,7-diphenyl-4,6-heptadien-3-one (L71, *m/z* 263.1422, R_t_: 14.53) (C), 1,7-diphenyl-5-hydroxy-4,6-heptadien-3-one, (L72, *m/z* 279.1371, R_t_:14.94) (D) and 1,7-diphenyl-1,6-heptadiene-3,5-dione (L73, *m/z* 277.1215, R_t_:15.01) (E), respectively.

**Table S1: Chemical structures of tentatively identified compounds in** **UHPLC-ESI- MS/MS.**

| **Sugars** | | | | | |
| --- | --- | --- | --- | --- | --- |
| 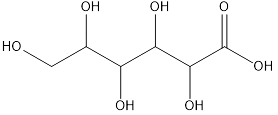 | | 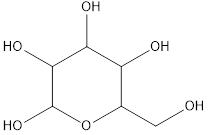 | | 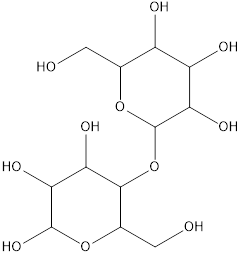 | |
| **(L1)** Gluconic acid | | **(L2)** Glucose /Galactose | | **(L3)** *O*-Hexosyl-hexose | |
| **Amino acids and nitrogenous compounds** | | | | | |
| 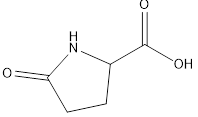 | | 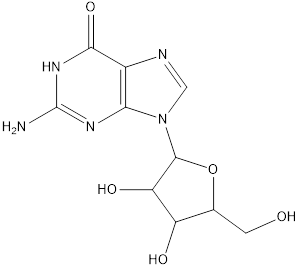 | 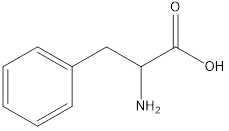 | | 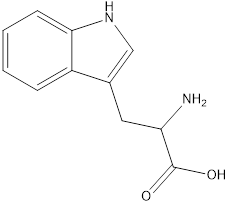 |
| **(L4)** Pyroglutamic acid | | **(L5)** Guanosine | **(L6)** Phenylalanine | | **(L7)** Tryptophan |
| **Organic acids** | | | | | |
| 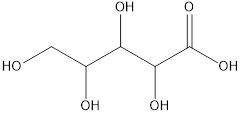 | | 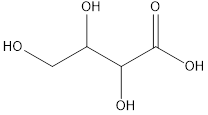 | 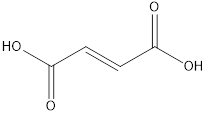 | | 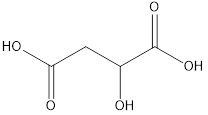 |
| **(L8)** Xylonic acid | | **(L9)** Threonic acid (R= OH) | **(L10)** Fumaric acid | | **(L11)** Malic acid |
| 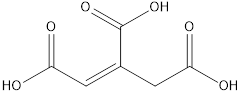 | 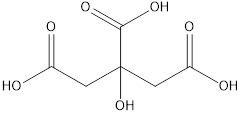 | | 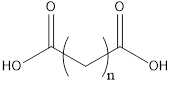 | | |
| **(L12)** Aconitic acid | **(L13)** Citric acid | | **(L14)** Suberic acid (n=6)  **(L15)** Azelaic acid (n=7) | | |
| **Phenolic acids** | | | | | |
| 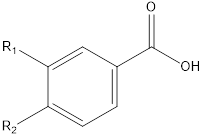 | | | 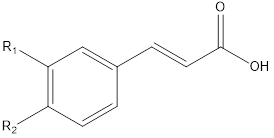 | | |
| **Hydroxybenzoic derivatives** | | | **Hydroxycinnamic derivatives** | | |
| **(L16)** Protocatechuic acid (R_1_=OH, R_2_= OH)  **(L17)** Vanillic acid hexoside (R_1_=OCH_3_, R_2_= *O*-hexose)  **(L18)** Hydroxybenzoic acid (R_1_=H, R_2_= OH)  **(L20)** Vanillic acid (R_1_= OCH_3_, R_2_= OH) | | | **(L19)** Coumaric acid (R_1_=H, R_2_= OH)  **(L21)** Ferulic acid (R_1_=OCH_3_, R_2_= OH)  **(L22)** Cinnamic acid (R_1_=H, R_2_=H) | | |

| **Flavonoids**  ***Flavonols*** | |
| --- | --- |
| **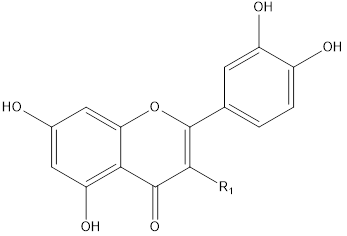** | **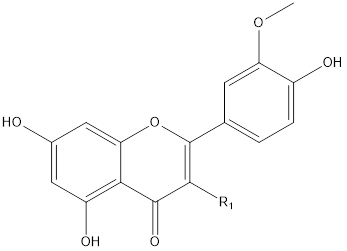** |
| **Quercetin derivatives** | **Isorhamentin derivatives** |
| **(L23)** Quercetin-*O*-rhamninoside (R_1_=*O*-rhamninose)  **(L25)** Rutin (R_1_=*O*-rutinose)  **(L26)** Quercetin-*O*-hexoside (R_1_=*O*-hexose)  **(L29)** Quercetin | **(L24)** Typhaneoside (R_1_=*O-*(2'',6''-di-*O*-rhamnose)-*O*-hexose)  **(L27)** Isorhamnetin-*O*-rutinoside (R_1_=*O-*rutinose)  **(L28)** Isorhamnetin-*O*-hexoside (R_1_=*O*-hexose) |
| **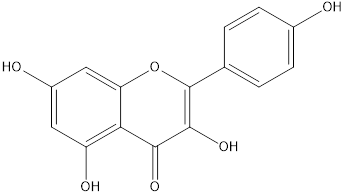** | 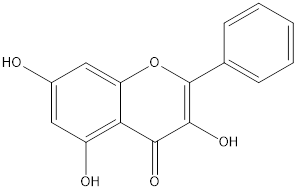 |
| **(L30)** Kaempferol | **(L31)** Galangin |
| **Flavonoids**  ***Flavanone*** |  |
| **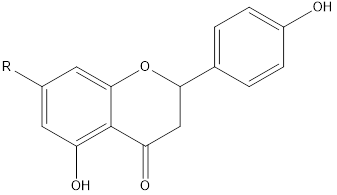** | **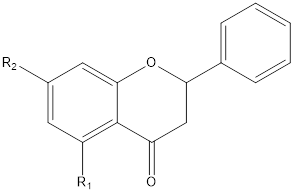** |
| **(L32)** Sakuranetin (R=OCH_3_)  **(L34)** Naringenin (R=OH) | **(L33)** Pinocembrin-*O*-hexoside (R_1_=OH, R_2_=*O*-hexose)  **(L36)** Pinocembrin (R_1_=OH, R_2_=OH)  **(L38)** Alpinetin (R_1_=OCH_3_, R_2_=OH) |
| **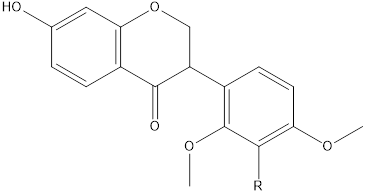** | **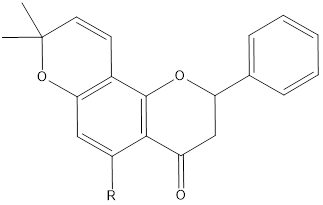** |
| **(L35)** Violanone (R=OH)  **(L37)** Sativanone (R=H) | **(L39)** Obovatin methyl ether (R=OCH_3_)  **(L40)** Obovatin (R=OH) |

| **Flavonoids**  ***Flavanonols*** | |  | |
| --- | --- | --- | --- |
| **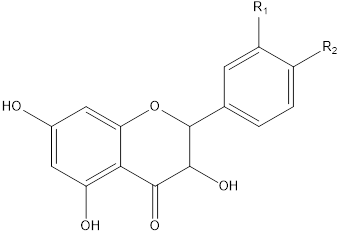** | | **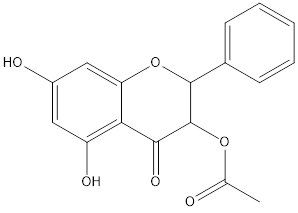** | |
| **(L41)** Dihydroquercetin (R_1_=OH, R_2_=OH)  **(L42)** Dihydroisorhamnetin (R_1_=OCH_3_, R_2_=OH)  **(L43)** Dihydrokaempferide (R_1_=H, R_2_= OCH_3_) | | **(L44)** Pinobanksin-*O*-acetate | |
| **Flavonoids**  ***Proanthocyanidin*** | | | |
| **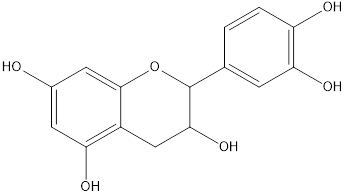** | **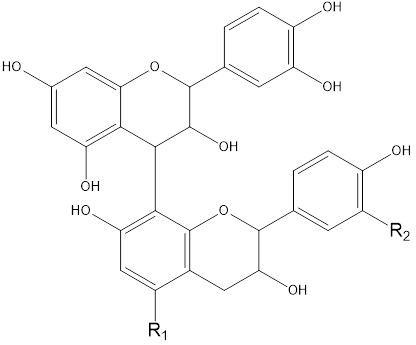** | |  |
| **(L46)** Catechin | **Dimer**  **(L45)** Catechin-(4→8)-catechin  (R_1_= OH, R_2_=OH)  **(L50)** Catechin-(4→8)-guibourtinidol  (R_1_= H, R_2_=H) | | **Trimer**  **(L47)** Catechin-(4→8)- catechin-(4→8)-catechin  (R_1_= OH, R_2_=OH)  **(L49)** Catechin-(4→8)- catechin-(4→8)-guibourtinidol  (R_1_= H, R_2_=H) |
| **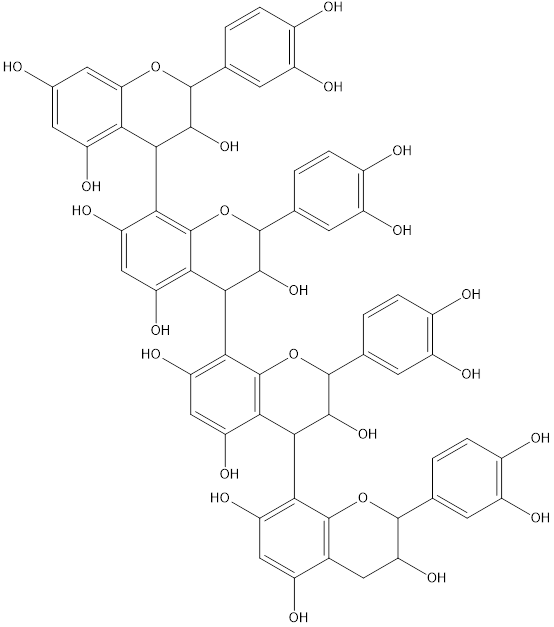** | | | |
| **Tetramer**  **(L48)** Cinnamtannin A2 | | | |

| **Chalcones and dihydrochalcones** | | | | |
| --- | --- | --- | --- | --- |
| **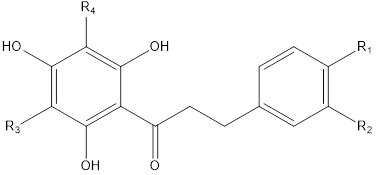** | **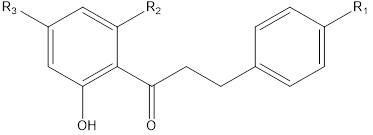** | | | 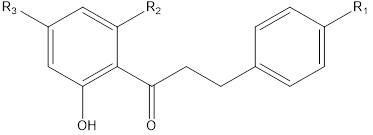 |
| **Dihydrochalcones glycosides** | **Dihydrochalcones** | | | **Chalcones** |
| **(L51)** Aspalathin  (R_1_=OH, R_2_=OH, R_3_=hexose, R_4_=H)  **(L52)** Phloretin-3',5'-di-C-hexoside  (R_1_=OH, R_2_=H, R_3_=hexose, R_4_=Hexose)  **(L53)** Nothofagin  (R_1_=OH, R_2_=H, R_3_=hexose, R_4_=H)  **(L54)** Deoxy phloretin-3',5'-di-C-hexoside  (R_1_=H, R_2_=H, R_3_=hexose, R_4_=hexose)  **(L55)** Deoxy phloretin-3'-C-hexoside  (R_1_=H, R_2_=H, R_3_=hexose, R_4_=H) | **(L56)** Phloretin  (R_1_=OH, R_2_=OH, R_3_=OH)  **(L57)** Sakuranetin dihydrochalcone  (R_1_=OH, R_2_=OH, R_3_=OCH_3_)  **(L58)** Pinocembrin dihydrochalcone  (R_1_=H, R_2_=OH, R_3_=OH)  **(L60)** Dihydroxy-dihydrochalcone  (R_1_=H, R_2_=H, R_3_=OH)  **(L64)** Dihydroxy−methoxy dihydrochalcone  (R_1_=H, R_2_=OH, R_3_=OCH_3_) | | | **(L59)** Flavokawain B  (R_1_=H, R_2_=OCH_3_, R_3_=OCH_3_)  **(L61)** 2',6'-dihydroxy-4,4'-dimethoxychalcone  (R_1_= OCH_3_, R_2_=OH, R_3_=OCH_3_)  **(L63)** Cardamonin  (R_1_= H, R_2_= OCH_3_, R_3_=OH) |
| **(L62)** De-*O*-methyl rotundaflavanochalcone (R=OH)  **(L65)** Rotundaflavanochalcone (R=OCH_3_) | | **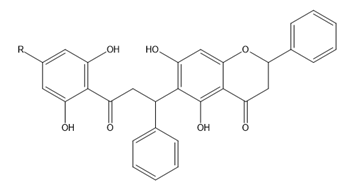** | | |
| **Calyxins** | |  | | |
| **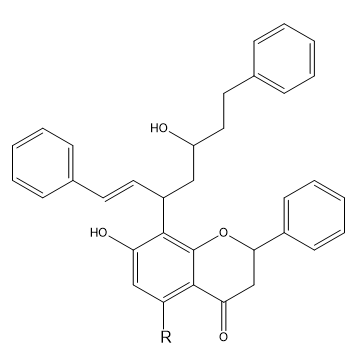**  **(L66)** Calyxin N or O (R=OCH_3_)  **(L68)** Calyxin P (R=OH) | | | **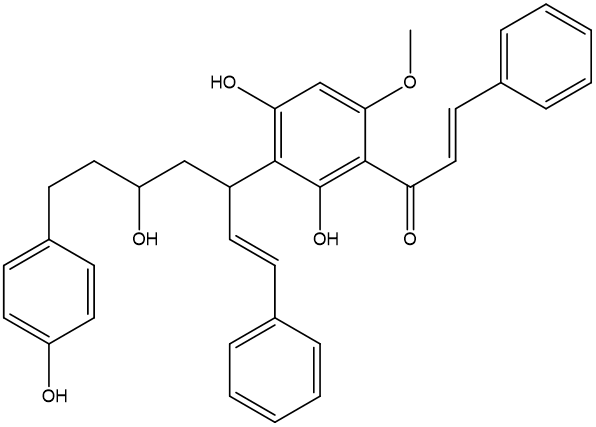**  **(L67)** Calyxin Q | |

| **Linear Diarylheptanoids** | | |  | | | | |
| --- | --- | --- | --- | --- | --- | --- | --- |
| **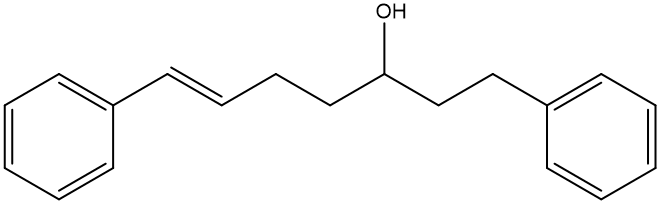** | | | **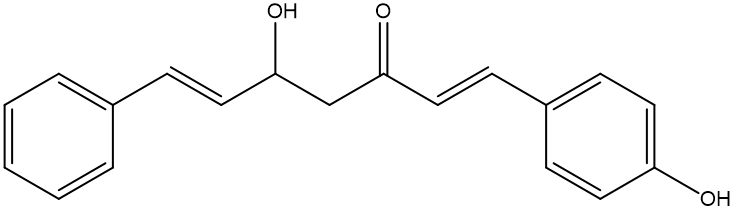** | | | | |
| **(L69)** 1,7-diphenyl-5-hydroxy-1-heptene | | | **(L70)** 5-hydroxy-1-(4-hydroxyphenyl)-7-phenylhepta-1,6-dien-3-one | | | | |
| **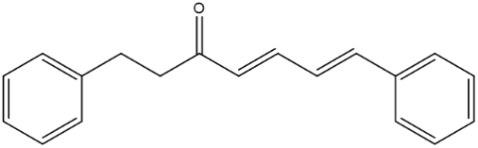** | | | **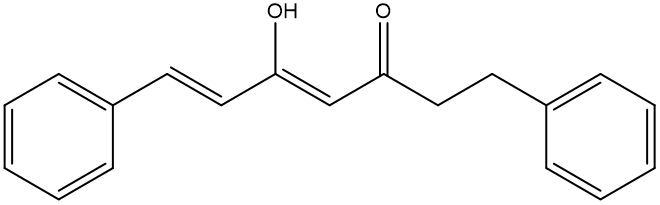** | | | **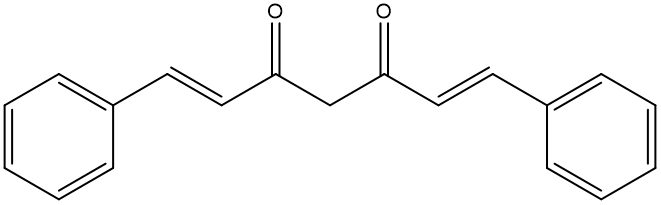** | |
| **(L71)** 1,7-diphenyl-4,6-heptadien-3-one | | | **(L72)** 1,7-diphenyl-5-hydroxy-4,6-heptadien-3-one | | | **(L73)** 1,7-diphenyl-1,6-heptadiene-3,5-dione | |
| **Kavalactones** | | | | | | | |
| **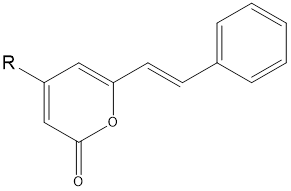** | | | | **(L74)** 4-hydroxy-6-styryl-2H-pyran-2-one (R=OH)  **(L75)** Desmethoxy yangonin (5,6-dehydrokawain)  (R=OCH_3_) | | | |
| **Miscellaneous** | | | | | | | |
| **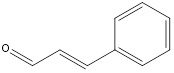** | **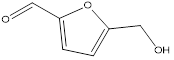** | | | **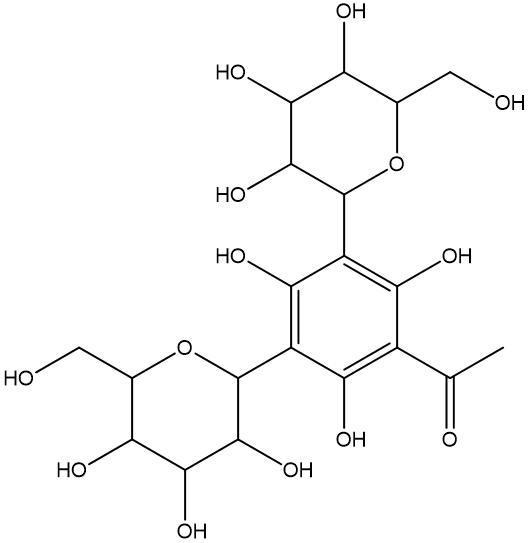** | | | **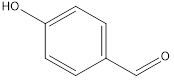** |
| **(L76)** Cinnamaldehyde | **(L77)** Hydroxy methyl-furaldeyde | | | **(L78)** 2ʹ,4ʹ,6ʹ-trihydroxy-acetophenone-3ʹ,5ʹ-di-C-hexoside | | | **(L79)** Hydroxybenzaldehyde |
| **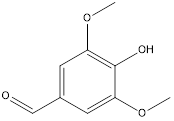**  **(L80)** Syringaldehyde | | **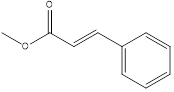**  **(L81)** Methyl cinnamate | | | **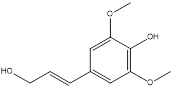**  **(L82)** Syringenin | | |
|  |  | | |  | | |  |
